# Spatial heterogeneity of soil phosphorus influencing bacterial functional adaptations in alkaline calcareous soils

**DOI:** 10.3389/fmicb.2025.1720323

**Published:** 2026-01-28

**Authors:** Saira Tabbasum, Mahreen Yahya, Munir Zia, Midrar ul Haq, Samina Anwar, Usama Azeem Khan, Naima Mahreen, Khansa Ejaz, Mika Tapio Tarkka, Sumera Yasmin

**Affiliations:** 1 Soil and Environmental Biotechnology Division, National Institute for Biotechnology and Genetic Engineering, College of Pakistan Institute of Engineering and Applied Sciences (NIBGE-C, PIEAS), Islamabad, Pakistan; 2 Department of Ecology and Agroecosytems, UFZ-Helmholtz-Centre for Environmental Research, Halle, Germany; 3 Research & Development Department, Sustainability & Innovation Division, Fauji Fertilizer Company Limited, Rawalpindi, Pakistan; 4 German Centre for Integrative Biodiversity Research (iDiv) Halle-Jena-Leipzig, Leipzig, Germany

**Keywords:** soil phosphorus heterogeneity, PSB, phosphate acquisition, edaphic profiling, bacterial ecophysiological adaptations, recursive partitioning regression analysis

## Abstract

To enhance sustainable soil fertility and efficient phosphorus (P) management, phosphate-solubilizing bacteria (PSB) play a central role in solubilizing soil mineral phosphorus by releasing organic acids and acidifying micro-niches. Thus far, the influence of spatial P heterogeneity on bacterial eco-physiological adaptations to P-limited, alkaline soils remains poorly understood. This study examined how soil edaphic factors vary across major wheat-growing regions, assessing their influence on the abundance and functional properties of culturable PSB. Soil available P was the strongest predictor of culturable bacterial abundance, with a threshold of *P* < 6.3 mg kg^–1^ dry soil driving major variations. At low P levels, organic matter played a key role, while at higher P levels, potassium (*K* ≥ 123) and pH further shaped bacterial abundance. Low-P soil PSB (L_*PSB*_) secreted elevated levels of organic acids such as malic, succinic, gibberellic and citric acid, but low levels of indole acetic acid. A clear trade-off was observed between P solubilization and growth-related traits: L_*PSB*_ invested more in acquiring resources (e.g., producing siderophores and organic acids) and less in synthesizing phytohormones. A net house study showed that L_*PSB*_ contribute to plant growth. Plants with 70% phosphate fertilization (P_70_) and PSB inoculation reached the yield levels comparable to those with 100% fertilization without the PSB, indicating the potential of PSB to reduce dependency on fertilizers. This was associated with a significant increase in wheat biomass (24.3%), yield (28.53%) and P use efficiency (31.66%) by L_*PSB*_ inoculation compared to the control P_70_. Our findings emphasize the importance of microbial functional plasticity in enhancing P use efficiency in P-limited soil, offering a basis for developing climate-smart bioformulations to improve sustainable crop productivity.

## 1 Introduction

Terrestrial ecosystems, particularly soil resources, are under increasing pressure due to rapid population growth and rising global demand for food. Intensive farming practices have exhausted the croplands by depleting soil organic matter and vital nutrients, such as phosphorus (P), which is essential for maintaining soil health and ecosystem functioning (Ahmed et al., 2025). As a key component of nucleic acids, phosphoproteins, coenzymes and phospholipids, P plays a central role in the metabolism of soil microbiota and in P biogeochemical cycling (Siciliano et al., 2014; Tian et al., 2021). The impact of P availability on soil microbial communities has been demonstrated across a wide range of terrestrial environments, including tropical forests in southern China (Liu et al., 2012) to tundra in northern Sweden (Rinnan et al., 2007) and Alaska (Koyama et al., 2013), as well as in agroforestry parklands in Burkina Faso (Gnankambary et al., 2008) and farmlands in Nyabeda, Kenya (Ehlers et al., 2010). Limited P supply can lead to multiple physiological and developmental impacts, including stunted growth, poor root development, and impaired photosynthesis and energy transfer (Wu W. et al., 2022), which has cascading effects on soil heterotrophs, predominantly microbial communities (Finkel et al., 2019). Despite its recognized role as a potent modulator of the soil microbiome (Cui et al., 2019), there are still significant gaps in our understanding of soil phosphorus-microbe interactions. This poses challenges for sustainable land management and ecological modeling (Čapek et al., 2016).

Soil bacteria are central to biogeochemical P cycling, acting as a source (remineralization) or sink (immobilization) of phosphate ions (Achat et al., 2010). Specifically, bacterial biomass transforms occluded and organic P by organic acids-mediated dissolution and enzymatic hydrolysis, ultimately adding diffusible P ions to the readily available P pool (Pang et al., 2024). Organic acids accelerate effective dissociation of phosphate (PO_4_^3−^) ions by regulating the soil P cycle, like sorption-desorption through protonation (H^+^), ion exchange, and chelation with calcium (Ca), aluminum (Al) and iron (Fe) (Adnan et al., 2019; An et al., 2022). The enhanced production of enzymes and organic acids for P solubilization can be linked to an increased metabolic rate (qCO_2_). This may result in higher carbon and energy consumption, and reflects the investment of microbes in P-mobilizing processes at the expense of growth or maintaining a low carbon use efficiency (Cui Y. et al., 2022; Feng and Zhu, 2019; Spohn and Chodak, 2015). Despite the extensive evidence that highlights the contributions of microbes to P mobilization and acquisition (Adnan et al., 2022; Ahmed et al., 2025; An et al., 2022; Bargaz et al., 2021; Wang Y. et al., 2023; Wu W. et al., 2022), the ecophysiological trade-offs that bacteria make, particularly between growth, organic acid production, P solubilization, and adaptation to low-P environments, remain poorly understood. Unraveling these trait trade-offs is important for a deeper understanding of soil phosphorus bioavailability and microbial ecophysiological adaptation.

The functional repertoire of soil bacteriome is significantly affected by edaphic factors and climate-induced soil heterogeneity (Liang et al., 2019; Wei et al., 2018; Xu et al., 2016). Earlier research syntheses have provided an empirical and theoretical basis for the idea that P cycling regulation is a collective trait of bacterial communities’ interactions with each other and with the edaphic factors (Gupta and Tiedje, 2024; Nielsen et al., 2010). One of the primary mechanisms adopted by soil bacteriome to enhance P availability is the production of organic acids (citric acid, oxalic acid, malic acid, gluconic acid, gibberellic acid, succinic acid and acetic acid). Due to that, a comprehensive understanding of how the production of these acids varies with P heterogeneity in soil is crucial to fully harnessing their potential to improve P bioavailability. It is also essential to understand how bacterial secretion of these acids varies across P gradients to improve nutrient management in agroecosystems.

To close these knowledge gaps, we investigated whether spatial P variability is a strong driver of bacterial ecophysiological traits and how soil P heterogeneity affects bacterial physiological responses and their contribution to plant P acquisition. To this end, we conducted a systematic analysis of 127 agricultural sites across Pakistan, integrating edaphic profiling, characterization of the culturable bacterial community, and regression partition analysis to identify the drivers of bacterial abundance. We also quantified the production of organic acids by PSB isolated from the spatially variable soils, and validated their contribution to plant P acquisition in a net house study. We hypothesized that spatial P variability directly modulates bacterial functional traits, resulting in cascading effects on P bioavailability and bacterial communities. Secondly, we hypothesized that the microbial communities in low-P soils exhibit enhanced organic acid secretion as an eco-physiological adaptation to mine recalcitrant P. We expected that combining edaphic variables with bacterial mechanistic trait measurements and plant-level responses would offer new insights into how microbial functional strategies emerge along phosphorus (P) gradients and potentially influence nutrient dynamics in agroecosystems.

## 2 Materials and methods

### 2.1 Study region and sampling regime

The study area encompasses two provinces of Pakistan, Punjab (31°08′49.6 “N 72°42′00.0″E) and Sindh (26°02′24.0 “N 68°42′36.0″E), with semi-arid to arid climatic conditions. Punjab is characterized by average annual temperature ranging from 5.2 to 39.8°C, precipitation 0.3–5.47 mm/day, relative humidity 37–69%, humidex 18–47°C, while Sindh has a warmer climate, with average temperatures ranging from 8.8 to 41.8°C, lower daily precipitation (0.03–2.87 mm), relative humidity of 45–69%, and a higher humidex (24–52°C) (WorldData.Info, 2025).

**FIGURE 1:**
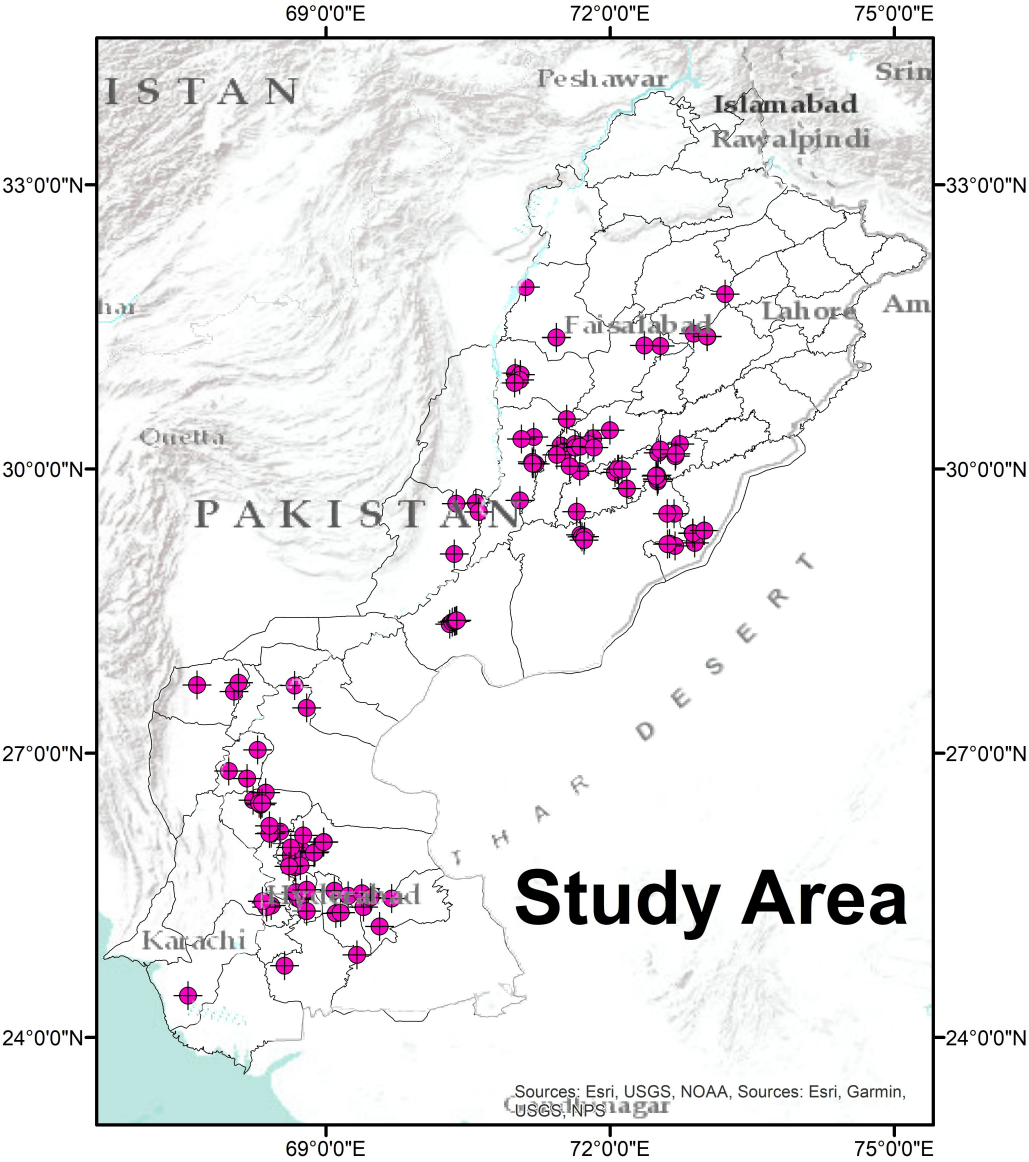
Geographical distribution of sampling sites. Soil sampling locations across Punjab and Sindh provinces of Pakistan are displayed using ArcGIS map tools. Soils were sampled from 127 agricultural sites, and 14 sites with contrasting soil available P were selected to study the effect of P availability on microbial functionality. Symbols representing closely located sites overlap on the map.

The sampling design was intended to support the evaluation of spatial phosphorus (P) heterogeneity, which refers to variations in soil phosphorus availability across geographically distinct wheat-growing areas in Punjab and Sindh. Soil samples were collected from 127 wheat fields, which were geographically distributed across a total of 22 major wheat-growing districts in Punjab and Sindh (Figure 1). Rhizospheric soil from 30 to 60 days old wheat plants was collected by carefully uprooting healthy plants, gently shaking off loose soil, and brushing the soil tightly adhering to the roots. We used this young age to investigate bacterial population during the early to mid-vegetative stage, which is critical for establishing root-associated microbiomes and early phosphorus acquisition. From each site, 3 samples were collected and mixed to form a composite sample. Soil subsamples were homogenized and sieved using a 2 mm mesh to remove roots and coarse debris, then transported to the lab and stored at 4°C for subsequent analyses. Among these sites, 14 locations with contrasting phosphorus (P) availability (high vs. low P) were selected based on a threshold of 15 mg/Kg. This threshold reflects the nationally recognized criterion for arable soils, wherein soils with P levels below 15 mg/kg (particularly up to 10 mg/kg) are considered low-P soils, while those exceeding 15 mg/kg are classified as high-P soils (Zia et al., 2021). The subset of 14 sites with contrasting soil available P was further used to study the effect of P availability on PSB’s functionality (Table 1; Supplementary Figure 2).

**TABLE 1:** Geographic location and available P content of sites with contrasting phosphorus (P) availability.

			High-P soils		Low-P soils	
ID	Province	Site location	GPS coordinates	Available *P* (mg/kg)	GPS coordinates	Available *P* (mg/kg)
1	Punjab	Bahawalnagar	29°19′15.1″N 72°52′25.4″E	20.45	29°20′54.5″N 72°59′43.8″E	4.25
2	Punjab	Bahawalpur	29°16′53.5″N 71°42′25.8″E	20.75	29°1705.9″N 71°43′55.6″E	9.50
3	Punjab	Multan	30°01′45.7″N 71°34′32.7″E	25.40	30°11′08.4″N 71°31′02.0″E	5.95
4	Punjab	Rahim Yar Khan	28°22′55.0″N 70°20′07.4″E	16.5	28°23′26.5″N 70°21′32.5″E	6.35
5	Punjab	Jhang	31°18′12.9″N 72°21′52.7″E	32.10	31°17′51.3″N 72°31′57.3″E	4.45
6	Punjab	Vehari	29°59′48.4″N 72°04′54.9″E	20.25	29°47′26.6″N 72°10′38.6″E	6.35
7	Punjab	Muzaffargarh	30°04′23.9″N 71°10′51.2″E	26.10	30°31′28.5″N 71°32′42.5″E	5.00
8	Punjab	Rajanpur	29°32′38.2″N 70°36′47.7″E	29.45	29°06′02.5″N 70°21′20.4″E	9.80
9	Sindh	Sanghar	25°48′41.5″N 68°43′55.0″E	17.80	25°47′39.3″N 68°40′33.0″E	6.90
10	Sindh	Shaheed Benazirabad	26°13′55.2″N 68°24′22.3″E	23.45	26°10′14.0″N 68°30′58.7″E	4.10
11	Sindh	Naushehro Feroze	27°01′56.3″N 68°16′44.8″E	15.50	27°01′56.3″N 68°16′44.8″E	7.85
12	Sindh	Mirpur Khas	25°32′48.3″N 69°05′29.8″E	16.3	24°52′03.0″N 69°19′47.7″E	4.35
13	Sindh	Hyderabad	25°26′03.5″N 68°19′46.4″E	29.15	25°22′20.4″N 68°22′29.6″E	9.30
14	Sindh	Khairpur	27°42′44.7″N 68°40′12.5″E	16.15	27°28′35.5″N 68°47′56.8″E	3.35

### 2.2 Chemical and microbial analyses of soils

To assess their edaphic properties, the soil samples were analyzed following standard procedures. Soil pH and electrical conductivity were analyzed using a 1:2.5 soil-water suspension measured with a calibrated digital pH and EC meter. Soil organic matter (SOM) was estimated following Walkley-Black chromic acid wet oxidation method (Bahadori and Tofighi, 2017). This involved oxidizing organic matter with a known excess of potassium dichromate (K_2_Cr_2_O_7_) in concentrated sulfuric acid (H_2_SO_4_), and quantifying the unreacted dichromate through back-titration with ferrous sulfate (FeSO_4_). Available P was measured by phosphomolybdate (molybdenum blue) method (Adelowo and Oladeji, 2016). Sodium bicarbonate (1:10, soil-solution) was used for extraction, followed by reaction with acidic molybdate reagent and formation of blue color complex. Colorimetric analysis was done spectrophotometrically (RMECO UV-6,000, Germany) at 880 nm. For available potassium (K) determination, ammonium acetate solution was used for extraction, and extract was further used for K estimation using flame photometer (Model Sherwood 420, Sherwood, United Kingdom) (Toth and Prince, 1949). Bacterial load of soil samples was measured by serial dilution method using LB agar plates.

### 2.3 Isolation, identification phenotypic characterization of PSB

Enrichment and isolation of PSB was done using National Botanical Research Institute’s Phosphate (NBRIP) media (Nautiyal, 1999) supplemented with tricalcium phosphate (TCP) as insoluble P source. Soil sample (2.5g) was added in 25mL of NBRIP broth and incubated at 28 ± 2°C on a rotary shaker at 150rpm for 72 h. Subsequently, 2.5mL suspension from the first enrichment was transferred to a fresh 25mL NBRIP broth and incubated under identical conditions for an additional 72 h to selectively enhance the population of PSB. Post-incubation, the soil suspension was serially diluted and spread on NBRIP agar plates, followed by 7 days incubation at 28 ± 2°C. The colonies with a clear halo zone around were selected and further purified by streaking on LB agar plates. Phosphorus Solubilization index (PSI) was calculated by following the formula stated by Amri et al. (2023). From each site, a single bacterial strain demonstrating the highest P solubilization potential was chosen for further characterization of plant growth-promoting (PGP) traits, the potential for phosphate solubilization and organic acids production.

The morphological characteristics of selected bacterial isolates were examined and they were grouped according to the partial 16S rDNA sequences. The bacterial cultures were grown overnight in LB broth at a shaking incubator at 28 ± 2 °C, followed by DNA extraction using CTAB method (Wilson, 2001). Polymerase chain reaction (PCR) was performed on the isolated bacterial DNA using (forward primer 5′-AGACTTTGATCCTGCTCAG-3′ and reverse primer 5′-AGGAGGTGATCCAGCCGCA-3′) (Ayyaz et al., 2016). For PCR amplification, initial denaturation at 95 °C for 60 s, followed by annealing at 53 °C for 30 sec, extension at 72 °C for 60 s, and a final extension at 72 °C for 10 min. The resulting product was run on agarose gel (1%), subsequently purified and sequenced commercially from Punjab Institute of Nuclear Medicine (PINUM), Faisalabad. The obtained sequences were blast at NCBI to identify bacterial strains and submitted to NCBI Gen Bank (Table 2).

**TABLE 2:** Selected phosphate-solubilizing bacteria (PSB) and their characterization for plant growth-promoting (PGP) traits.

Site ID	Location	Isolated PSB	Colony morphology	Accession No.	^1^IAA	^2^Zn(SI)	^3^Siderophore	^4^EPS
1	Bahawal-nagar	*Acinetobacter lactucae*: HST3	Circular, off-white, entire, raised, smooth, shiny.	PV688309	**+**	5.63	**−**	**−**
		*Enterobacter cloacae*: LST5	Circular, white, entire, umbonate, gummy, shiny.	OR945743	**+**	4	**+++**	**−**
2	Bahawal-pur	*Franconibacter daqui*: HST32	Circular, yellow, entire, umbonate, smooth, dull.	OR945762	**+**	3.1	**++**	**+**
		*Klebsiella quasivariicola*: LST36	Circular, off white, entire, convex, smooth, shiny, gummy.	OR945765	**−**	5.9	**−**	**−**
3	Multan	*Enterobacter cloacae*: HST37	Circular, off white, entire, pulvinate, smooth, shiny.	OR945766	**++**	5	**−**	**−**
		*Acinetobacter baumannii*: LST1	Circular, white, entire, raised, smooth, shiny.	OR945742	**+**	4.6	**+**	**−**
4	Rahim Yar Khan	*Acinetobacter calcoaceticus*: HST22	Circular, yellow, entire, umbonate, dull, gummy.	OR945755	**+**	4.9	**−**	**−**
		*Klebsiella quasivariicola*: LST11	Circular, off-white, entire, raised, smooth, shiny, gummy.	OR945747	**+**	5.2	**+++**	**−**
5	Jhang	*Pantoea dispersa*: HST9	Circular, yellow, entire, flat, smooth, shiny.	OR945745	**++**	3.75	**−**	**−**
		*Pantoea dispersa*: LST6	Circular, yellow, entire, convex, smooth.	OR945744	**+**	5	**+++**	**−**
6	Vehari	*Citrobacter youngae*: LST13	Irregular, off-white, undulate, flat, gummy, rough.	OR945749	**+**	4.75	**−**	**−**
		*Klebsiella pneumoniae*: HST4	Circular, white, entire, umbonate, gummy, shiny	PV688310	**+**	5.88	**−**	**−**
7	Muzaffar-garh	*Acinetobacter pittii*: HST23	Circular, off white, entire, raised, smooth, shiny.	OR945756	**+**	7.2	**−**	**−**
		*Enterobacter cloacae*: LST10	Irregular, white, undulate, raised, smooth, shiny.	OR945746	**−**	4.88	**+++**	**−**
8	Rajanpur	*Acinetobacter calcoaceticus*: HST34	Circular, cream, entire, convex, smooth, dull.	PV688308	**++**	2.3	**−**	**−**
		*Acinetobacter lactucae*: LST30	Circular, white, entire, convex, wrinkled, shiny.	OR945760	**+**	5	**+**	**−**
9	Sanghar	*Acinetobacter lactucae*: HST24	Circular, off white, undulate, convex, smooth, glistening.	OR945757	**+**	4.9	**+**	**−**
		*Acinetobacter pittii*: LST20	Circular, off white, entire, convex, smooth, glistening.	OR945754	**−**	5.33	**−**	**−**
10	Shaheed Benazira-bad	*Klebsiella Africana*: HST35	Circular, off white, entire, convex, smooth, shiny, gummy.	OR945	**+**	6.8	**−**	**−**
		*Enterobacter cloacae* subsp. *Dissolvens*: LST17	Circular, off white, entire, convex, smooth, glistening.	OR945752	**−**	4.4	**+++**	**−**
11	Nausheh-ro Feroze	*Acinetobacter calcoaceticus*: HST19	Circular, yellow, entire, umbonate, smooth, shiny, gummy.	OR945753	**+**	3.76	**−**	**−**
		*Enterobacter cloacae*: LST12	Circular, off-white, entire, pulvinate, dull, smooth.	OR945748	**−**	3.75	**−**	**−**
12	Mirpur Khas	*Acinetobacter geminorum*: HST29	Circular, white, entire, convex, wrinkled, dull.	OR945759	**+**	6	**+**	**−**
		*Acinetobacter pittii*: LST16	Circular, off white, entire, umbonate, smooth, shiny.	OR945751	**−**	6.125	**+**	**−**
13	Hydera-bad	*Enterobacter cloacae* subsp. *Dissolvens*: HST28	Irregular, off-white, smooth, shiny, flat, gummy.	OR945758	**+**	2.4	**+++**	**−**
		*Enterobacter bugandensis*: LST14	Circular, off-white, lobate, umbonate, rough, gummy	OR945750	**+**	8.33	**++**	**−**
14	Khairpur	*Citrobacter fruendii*: HST18	Circular, off white, entire, umbonate, smooth.	PV688307	**++**	6.25	**+**	**−**
		*Acinetobacter calcoaceticus*: LST31	Circular, white, entire, convex, wrinkled, dull	OR945761	**−**	4.3	**−**	**−**

^1^Indole Acetic Acid (IAA) production: **−** indicates no production, + indicates moderate production, ++ indicates high production, +++ exhibits relatively higher production of siderophores.

^2^Plate assay was used to assess zinc solubilization ability of isolated strains; SI stands for solubilization index.

Characterization of PSB for PGP traits was done using established protocols. Indole-3-acetic acid production was quantified using Salkowski method (Gordon and Weber, 1951). Bacterial cultures were grown in LB broth supplemented with 0.01% tryptophan and incubated on a shaker at 110 rpm for 7 days. To quantify IAA, 100 μL of each culture was transferred to a microtiter plate and thoroughly mixed with 100 μL of Salkowski’s reagent (0.5 M FeCl_3_: 1 mL; concentrated H_2_SO_4_: 30 mL; distilled water: 50 mL). After incubation at room temperature for 30 min, the development of a pink coloration indicated the presence of IAA.

Siderophore production was assessed using Chrome Azurol S (CAS) agar medium (Schwyn and Neilands, 1987). Pure bacterial cultures were streaked on Chrome Azurol S (CAS) agar plates and incubated at 28 ± 2 °C for 3–4 days. where the development of a pink halo zone around colonies indicated siderophore production activity. Following incubation, the plates were examined for the development of pink–purple coloration around bacterial colonies, indicative of siderophore production.

Zinc (Zn) solubilization was determined on Tris-minimal salts medium (composition per liter: dextrose 10 g (NH_4_)_2_SO_4_ 1 g, KCl 0.2 g, K_2_HPO_4_ 0.1 g, MgSO_4_⋅7H_2_O 0.2 g, ZnO 1 g, agar 2%) supplemented with zinc oxide as insoluble Zn source and incubated at 30 ± 2 °C for 24 h. The formation of clear halo zones around bacterial colonies demonstrated Zn solubilization activity. The solubilization index (SI) was calculated as described by Fasim and Ahmed (Fasim et al., 2002).

Exopolysaccharide (EPS) production was evaluated on Congo red agar medium (Congo red dye added in brain heart infusion (BHI) agar medium supplemented with 5% sucrose), where the appearance of brown to black pigmentation of bacterial colonies indicated EPS production (Moghannem et al., 2017).

### 2.4 Phosphorus solubilization and quantification of organic acids

Phosphate-solubilization and organic acid production of bacterial isolates were studied in an *in vitro* assay. A single colony of each isolate was inoculated in NBRIP broth medium (containing TCP as insoluble P source) and incubated for 7 days at 28 ± 2°C and 180 × *g*. Post-incubation, bacterial cultures were centrifuged at 4,000 rpm for 10 min at 4°C to obtain cell-free supernatants. Soluble P in the supernatants was quantified using the molybdenum blue method, and then spectrophotometer was used to measure the absorbance of samples at 880 nm (Murphy and Riley, 1962).

For the analysis of organic acids produced by the inoculated bacteria, the supernatants used for P quantification were filtered using 0.2 μm syringe filters and subjected to High-Performance Liquid Chromatography (HPLC; Agilent 1,200 Series; Agilent Technologies, Santa Clara, CA, United States) equipped with a C-18 column. The mobile phase consisted of methanol and phosphate buffer (90:10 v/v, pH 2.7), with a flow rate of 1 ML/min and detection at 210 nm. Organic acids such as acetic, citric, gluconic, gibberellic, malic, succinic and oxalic acids were identified and quantified by comparing retention times and peak areas with known standards (Yahya et al., 2021).

### 2.5 *In planta* evaluation of phosphorus mobilization in plant-soil environment and impact on wheat growth and productivity

#### 2.5.1 Experimental setup

To evaluate the impact of PSB isolated from low-P and high-P soils on soil P availability and on wheat growth, a pot experiment was conducted at National Institute for Biotechnology and Genetic Engineering (NIBGE) (31°23′45.1 “N 73°01’34.1”E), Faisalabad, under net house conditions in wheat growing season (November to April 2022-23). Soil was collected from the upper layer (0–20 cm) of agricultural field with wheat-rice rotation located in NIBGE, Faisalabad, Pakistan. The soil had loamy texture with 8.05 pH, 0.9% organic matter, 9.03 mg available P kg^–1^, 118 mg exchangeable potassium kg^–1^ and 112.08 mg mineral nitrogen kg^–1^. The soil was air dried, homogenized, sieved and then autoclaved to avoid potential effect of resident soil bacterial community, and to evaluate the specific functional contributions of the inoculated bacterial strains. Conducting the experiment in sterilized soil provides a controlled environment in which to gain mechanistic insights into plant–microbe interactions. However, these findings only serve as proof of concept for future studies, including field trials. The earthen pots (circumference = 102 cm, diameter = 30 cm, height = 36 cm) were filled to two-thirds of their capacity with autoclaved soil and watered to attain soil field capacity to assure good germination.

The experiment was arranged in a completely randomized design with the following four treatments included Control-1 (T1): 100% of the recommended dose of chemical fertilizer (100% CF); Control-2 (T2): 70% of the recommended dose of chemical fertilizer (70% CF); Inoculated-L_*PSB*_ (T3): inoculation of PSB isolated from low P soils + 70% CF; Inoculated-H_*PSB*_ (T4): inoculation of PSB isolated from high P soils + 70% CF. The recommended dosage of N (150 kg ha^–1^) and P (100 kg ha^–1^) was applied in form of urea and diammonium phosphate, respectively (Santoyo et al., 2012). The chemical fertilizer DAP was applied at the time of sowing, while urea was split into three doses; first at sowing, second and third at first and second irrigation, respectively.

The contribution of PSB in P availability and improving wheat growth was corroborated by *in planta* study in which the selection protocol prioritized bacterial strains demonstrating superior phenotypic characteristics, higher P solubilization potential, and diversified organic acid production. The selection criteria were then extended to maximize phylogenetic diversity by selecting PSB from different genera. Taking this in account, *Acinetobacter lactucae* LST30, *Pantoea dispersa* LST6 and *Enterobacter* sp. LST14 were selected to develop Consortium-1 (L_*PSB*_), while *Acinetobacter geminorum* HST29, *Citrobacter* sp. HST18 and *Franconibacter* sp. HST32 were chosen to develop Consortium-2 (H_*PSB*_). Before developing the microbial consortium, the compatibility of the selected PSB strains was assessed using a well diffusion assay. Each strain was first grown individually in LB broth. Then, 100 μL of the culture was spread onto LB agar plates. Subsequently, 30 μL of the remaining strains were added to the wells and the plates were incubated at 28 ± 2 °C for 72 h. The absence of halo zones around the wells indicated that the strains were compatible with each other and suitable for consortium development.

To develop the consortium, the following bacterial strains were inoculated in LB broth and kept overnight on a shaker at 140 rpm (30 ± 2 °C): *Acinetobacter lactucae* LST30, *Pantoea dispersa* LST6 and *Enterobacter* sp. LST14, *Acinetobacter geminorum* HST29, *Citrobacter* sp. HST18 and *Franconibacter* sp. HST32. The cultures of the bacterial strains (LST6, LST14 and LST30) were mixed at an equal volume (30 mL each) to develop consortium 1 (L_*PSB*_), while the cultures of the other strains (HST18, HST29 and HST32) were used to develop consortium 2 (H_*PSB*_). Seeds of recommended wheat variety: Akbar-2019 were sterilized using sodium hypochlorite solution (2–3%) and then washed thrice with autoclaved distilled water. The sterilized seeds were then submerged in bacterial consortium (concentration of 1 × 10^8^ CFU mL^–1^) for half an hour. For the control treatment, the seeds were soaked in LB broth.

#### 2.5.2 Evaluation of bacterial inoculation on wheat growth, yield and P content

To assess the impact of bacterial inoculation on wheat growth at different growth stages (35 days after germination (DAG), 70 DAG and harvest stage) wheat plants were uprooted (three plants for each treatment) and studied for different growth parameters, including root length, shoot length, fresh weight and dry weight. At harvest stage, plant biomass, grain yield and 1,000-grain weight were measured. During the vegetative stage, chlorophyll content in wheat plants was measured using a SPAD meter to non-destructively assess the physiological impact of a specific treatment. To measure aboveground P accumulation in wheat straw and grains, P content of plants harvested at 35 and 70 DAG and grains at harvest stage was estimated by Olsen method (Olsen, 1954). Phosphorus use efficiency (PUE) was calculated using following formula (Rasul, 2016; Sultana et al., 2021):


$$Puseefficiency\left(\%\right)=\frac{P\,u\,p\,t\,a\,k\,e\,i\,n\,g\,r\,a\,i\,n\,s\,\left(g/p\,o\,t\right)}{P\,a\,p\,p\,l\,i\,e\,d\,p\,e\,r\,p\,o\,t\,\left(g/p\,o\,t\right)}\times 100$$

#### 2.5.3 Detection of inoculated PSB and evaluation of their impact on soil P

At 35 DAG, rhizospheric soil of wheat plants was taken and stored at 4°C. The inoculated bacteria were re-isolated using serial dilution plate spread method from rhizospheric wheat soil. Bacterial population size was determined by colony forming units and specific strains identified by visually comparing their pure colony morphology, as reported by Yasmin et al., (2016). Re-isolated strains’ DNA was extracted by CTAB method and subjected to BOX-PCR (using the primer 5′ CTACGGCAAGGCGACGCTGACG 3′) to confirm the presence of the inoculated strains at the DNA level (see Supplementary Figure 3; Suleman et al., 2018). Soil available P was measured by phosphomolybdate (molybdenum blue) method, using sodium bicarbonate extraction (Adelowo and Oladeji, 2016).

### 2.6 Data processing and analysis

#### 2.6.1 Spatial variability mapping

**FIGURE 2:**
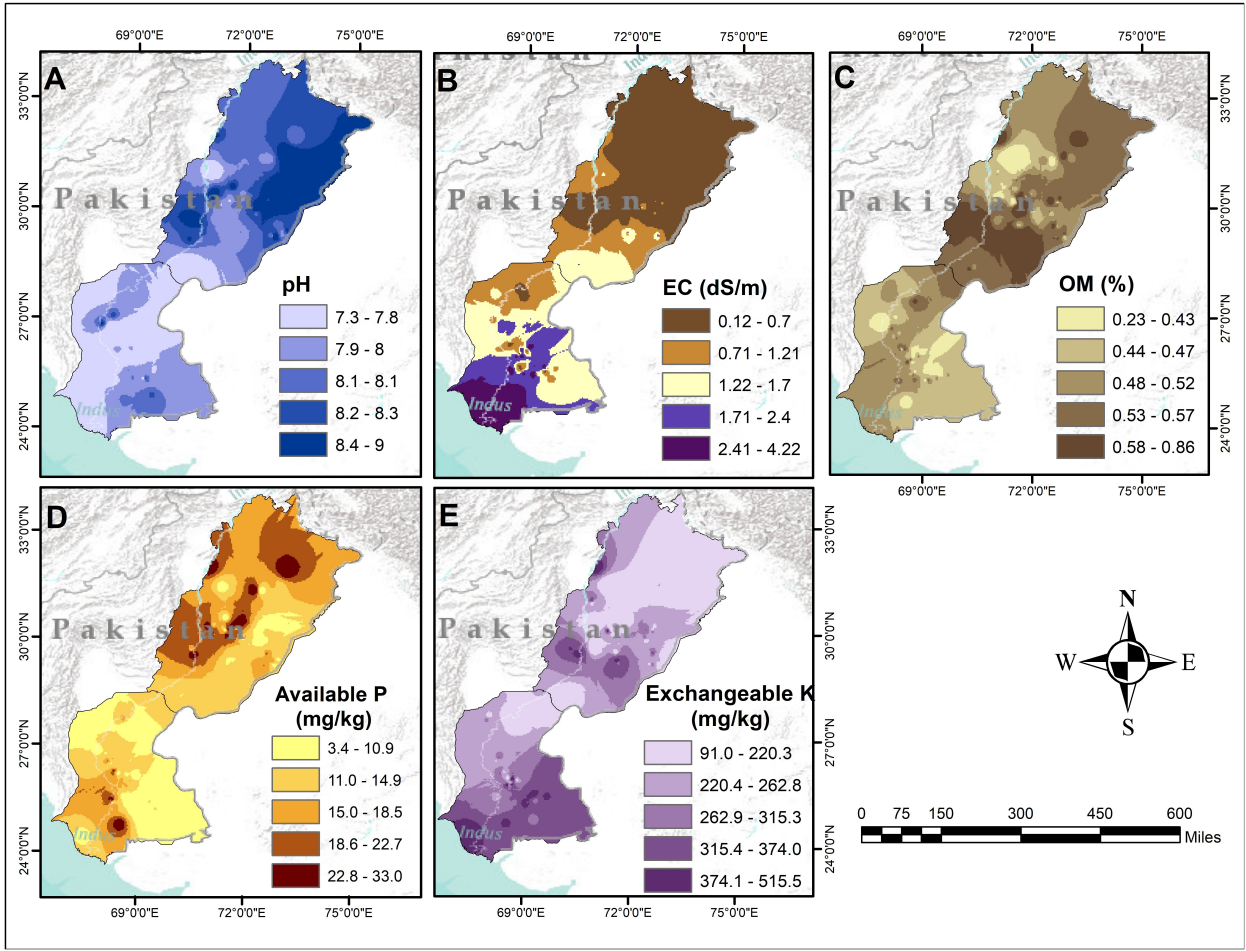
Spatial distribution patterns of edaphic factors across Punjab and Sindh provinces of Pakistan. The edaphic factors are following: soil pH **(A)**, electrical conductivity: EC **(B)**, organic matter: OM **(C)**, available phosphorus: P **(D)** and exchangeable potassium: K **(E)**.

The Inverse Distance Weighing (IDW) technique was used for interpolation to estimate the soil properties at unsampled locations based on soil parameters measured at sampling sites, across Punjab and Sindh provinces of Pakistan (Figure 2). The IDW method estimates values at unknown locations by calculating a distance-weighted average of nearby known points (Bel-Lahbib et al., 2023).

#### 2.6.2 Statistical analysis

For the regression analyses, the complete dataset was first analyzed and outliers were identified for each soil parameter (pH, electric conductivity, organic matter, P and K) using the interquartile range (IQR) method. Values falling outside 1.5 × IQR from the quartiles were flagged as extreme. These outliers were visualized in scatter plots against log-transformed CFU counts to assess their distribution. The flagged data were then removed for subsequent analyses, with a refined data set of 84 sites being used for further analysis. Regression analyses were performed using RStudio software (version 2024.12.0+467). To recursively partition bacterial log CFU data by edaphic properties, including soil pH, EC, organic matter, available P and extractable K, regression tree analysis was performed on the dataset (84 sites) using recursive partitioning and regression trees using the rpart package. Due to complex non-linear interactions among bacterial CFU and edaphic factors, a single linear model application was difficult. Thus, this approach was used to partition the data into smaller sets to study the interactions more effectively. For each site, the mean values of the replicate measurements were used for analysis. Based on the regression tree, the root node value (*P* < 6.3) was considered as threshold for regression analysis among different edaphic parameters and bacterial CFU of the selected sites with contrasting soil P availability, using a generalized additive model. The three replicates from each site were treated independently to account for spatial non-independence.

Organic acid concentration profiles of isolates were visualized by heatmap, generated using *pheatmap* package in R. To analyze data collected from pot experiment at different growth stages, analysis of variance (ANOVA) was performed using Statistix 10 software (Analytical Software, Tallahassee, United States). Prior to performing ANOVA, the assumptions of normality and homogeneity of variance were assessed. The homogeneity of variances was evaluated using Bartlett’s test. Significant differences among treatments were assessed by least significant difference (LSD) test at 5% significance level.

## 3 Results

### 3.1 Spatial distribution patterns of edaphic properties

Geospatial interpolation of key soil properties including pH, electrical conductivity (EC), organic matter (OM), available phosphorus (P) and exchangeable potassium (K) revealed variation in these parameters across the investigated area (Figure 2). The pH in most regions fell within the moderately to strongly alkaline range (7.3–9.0) (Figure 2A). Most of the Sindh province showed a pH value of between pH 7.3 and 8.1, while Punjab province exhibited higher values, ranging from pH 7.3 to 9.0. The strongly alkaline soils may pose challenges for nutrient availability, particularly phosphorus. Electrical conductivity (EC) ranged from 0.12 to 4.22 dS/m, with majority of Punjab’s soils exhibiting non-saline to moderately saline range (0.12–1.21 dS/m) (Figure 2B). However, pockets of high salinity areas ranging from 1.71 to 4.22 dS/m were observed along the Indus River delta and coastal areas in southern Sindh. Relatively low levels of organic matter (OM) were found across the whole study area, ranging from 0.23 to 0.86%. This is below the threshold value of 2% (Figure 2C). Extremely low OM content reflects intensive cultivation and high decomposition rates under arid conditions. The availability of P varied markedly across different regions, ranging from 3.4 to 33.0 mg/kg (Figure 2D). Extremely low levels of available P (3.4–10.9 mg/kg) were found across vast areas in Sindh and Punjab, with the exception of small pockets with high levels of P (18.6–33.0 mg/kg) (Figure 2D). Low P availability could be attributed to high pH and calcium carbonate content that limit P solubility. Exchangeable potassium (K) concentrations range from 91.0 to 515.5 mg/kg (Figure 2E). Northern regions show relatively low but sufficient K content (91.0–220.3 mg/kg), while southern and southwestern regions exhibit higher K levels (315.4–515.5 mg/kg), likely due to mica clay-rich mineralogy of the soils. The interpolated maps revealed significant spatial heterogeneity of soil variables, which could have a substantial influence on the microbial functional landscape.

### 3.2 Empirical relationships between bacterial abundance and edaphic factors

To understand how edaphic parameters (including pH, EC, OM, available P and exchangeable K) influence culturable bacterial population, regression analysis was performed for soils sampled from spatially dispersed areas across Pakistan. The bacterial population appeared to be driven by complex interactions of multiple edaphic properties (Cheng et al., 2021) that could not be assembled using a single linear model. To unravel these interactions, recursive partitioning was done using regression tree analysis, which depicted available P as the most influential factor affecting culturable bacteriome (Supplementary Figure 1), suggesting *P* < 6.3 mg kg^–1^ dry soil is a primary driver of variation in the dataset. At low P levels, OM plays a significant role with a threshold of 0.44. Furthermore, internal nodes indicated a decrease in bacterial colony forming unit (CFU) at low OM and very low P (*P* < 4.7), while at higher EC (≥ 0.6) increase in CFU potentially reflects better nutrient availability. At higher P availability, potassium is the second key partitioning variable with a threshold of K ≥ 123. In soils with higher P and K availability, bacterial abundance is further influenced by EC < 0.42, followed by pH levels (<7.9 and ≥ 8.8). Leaf nodes exhibited high bacterial count at very high pH (≥8.8), which suggests that bacterial communities shift toward copiotrophic life strategies in nutrient-rich soils (K ≥ 123) (Malik et al., 2018).

**FIGURE 3:**
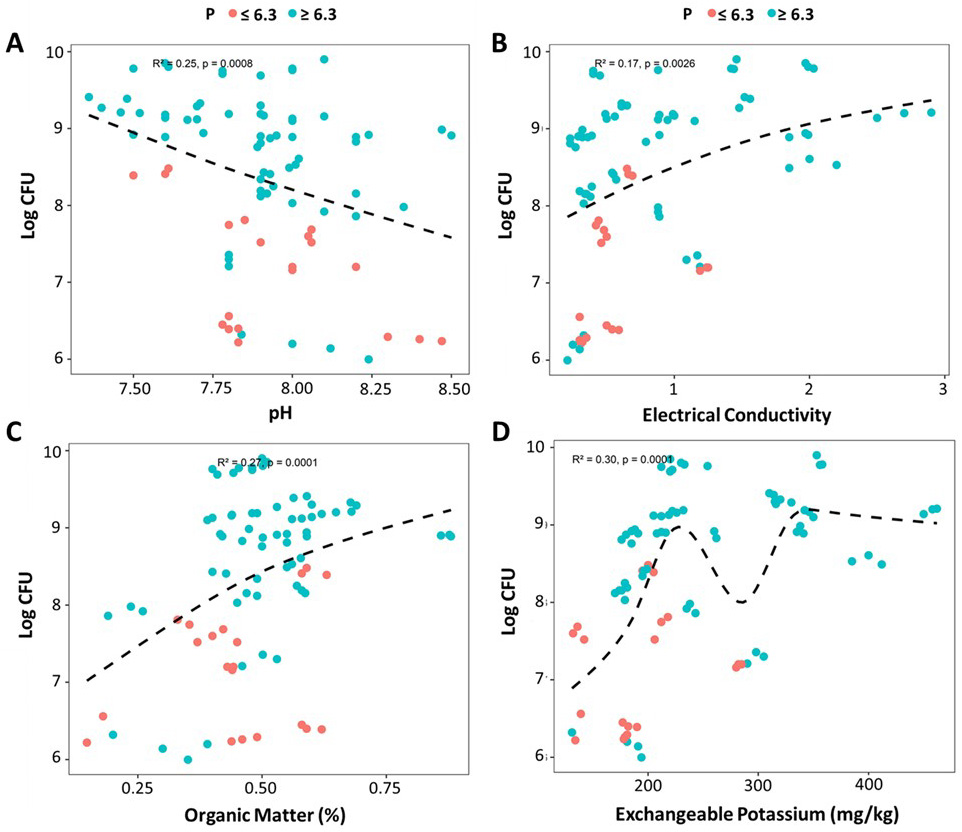
Relationship between edaphic parameters and soil bacterial CFU. Regression trends of soil pH **(A)**, electrical conductivity **(B)**, organic matter (OM) **(C)**, exchangeable potassium (K) **(D)** with bacterial CFU at a *P*-value threshold of 6.3 across selected Low-P and High-P soils. Data from all 14 locations (28 soil sampling sites with three replicates at each site) are presented here as independent points (blue circles: *P* ≥ 6.3, red circles: *P* < 6.3).

Regression analyses were performed at threshold P (6.3) among bacterial abundance and different soil physicochemical properties for the selected P contrasting soil samples. Soil pH significantly affected bacterial population in a negative correlation, with highest microbial counts at pH = 7.5–8.15 and a subsequent decrease further (Figure 3A). It is probable to associate higher CFU in relatively low pH regions with high P availability (>6.3). We also observed that in these soils, increased bacterial abundance was found in weak-to-moderate positive correlation with EC (Figure 3B), suggesting higher nutrient or ion concentration at higher EC, which could be correlated to high P availability. Further, bacteria were more abundant in soils with higher organic matter (Figure 3C), providing corroborating evidence that richer organic matter soils provide more carbon and energy sources, enhancing microbial growth (Dittmann et al., 2025). A non-linear positive correlation was found between exchangeable soil K and bacterial CFU, with highest count at *K* = 200–350 mg/kg (Figure 3D). This indicates that microbial abundance may be optimized at specific K concentrations, possibly due to interactions with other nutrients where K contributes to microbial growth, but only up to a certain level.

### 3.3 Uncovering the role of bacterial physiology in soil P availability

#### 3.3.1 Determination and phenotypic characterization of 28 selected PSB strains

Phosphate-solubilizing bacteria (PSB) were isolated from selected Low-P and High-P soils. The PSB with highest solubilization efficiency, as determined by P solubilization index (PSI), were selected for further characterization of their phenotypic and plant growth-promoting (PGP) traits. The 28 selected strains, which were either isolated from low-P (L_*PSB*_) or high-P (H_*PSB*_) soils, were further characterized by partial 16S rRNA sequencing (Table 2). Based on the analysis of the 16S rDNA sequences, *Acinetobacter* and *Enterobacter* strains were the most abundant. Indole acetic acid production was relatively high in H_*PSB*_, including *Enterobacter* sp. HST37, *Pantoea* sp. HST9, *Acinetobacter* sp. HST34 and *Citrobacter* sp. HST18 (Table 2). Conversely, most of the L_*PSB*_ exhibited high siderophore production as evidenced by strains *Enterobacter* sp. LST5, *Klebsiella* sp. LST11, *Pantoea* sp. LST6, *Enterobacter* sp. LST10, *Enterobacter* sp. LST17 and *Enterobacter* sp. LST14 (Table 2), corroborating siderophore’s role in enhancing PSB survival and function in the rhizosphere (Pan and Cai, 2023). Zinc (Zn) solubilization activity was detected for all PSB strains, with Zn solubilization indices (SI) ranging from 2.3 to 8.33 (Table 2). The highest SI was exhibited by *Enterobacter* sp. LST14 (8.33), followed by *Acinetobacter* sp. HST23 (7.2) and *Klebsiella* sp. HST35 (6.8). In contrast to Zn solubilization, the PSB strains exhibited negative results for exopolysaccharide (EPS) production, except for *Franconibacter* sp. RST32 (Table 2).

#### 3.3.2 *In vitro* quantification of tricalcium phosphate solubilization

**FIGURE 4:**
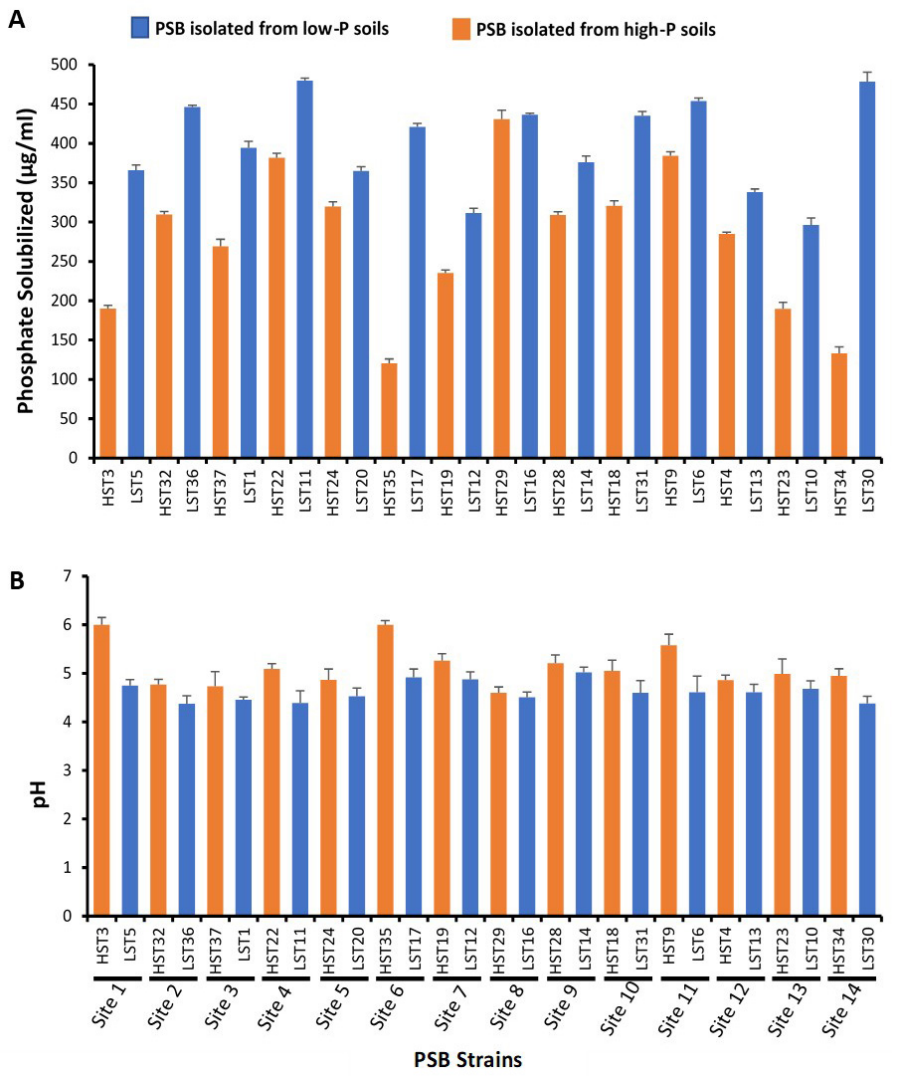
*In vitro* Phosphate Solubilization and pH Changes by PSB. **(A)** P Solubilization by isolated PSB in NBRIP broth supplemented with TCP as insoluble P source at 7 Days Post Inoculation (DPI). **(B)** pH of medium having TCP at 7 DPI (initial pH was 7 ± 0.2).

In NBRIP broth medium containing tri-calcium phosphate (TCP) as insoluble P source, P solubilization activity was exhibited by PSB strains with a concomitant decrease in pH (Figure 4). At 7 DPI, the range of solubilized P was 296.2–480.0 μg/mL and 120.5–431.0 μg/ml for L_*PSB*_ and H_*PSB*_, respectively, with a corresponding pH drops from 7 to 4.39 and 4.60. Overall, the PSB isolated from low-P soils exhibited higher P solubilization potential, with a maximum P release (480.0 μg/mL) by *Klebsiella quasivariicola* LST11, followed by *Acinetobacter lactucae* LST30 (478.6 μg/mL) and *Pantoea dispersa* LST6 (454.0 μg/mL) (Figure 4A). The P solubilization activity can be correlated with pH dynamics; acidification of the medium accelerated the solubilization mechanism, as evidenced by subsequent declines in the pH to 4.39, 4.38, and 4.61 by LST11, LST30, and LST6, respectively (Figure 4B). Conversely, the P solubilization potential of PSB isolated from high-P soils was found to be relatively low. Solubilized P was noted at 431.0, 384.1, 381.7 μg/ml for strains *Acinetobacter* sp. HST29, *Pantoea* sp. HST9 and *Acinetobacter* sp. HST22, respectively, with a concomitant decrease in pH from 7.0 to 4.6, 5.58, and 5.09.

#### 3.3.3 Organic acid production

**FIGURE 5:**
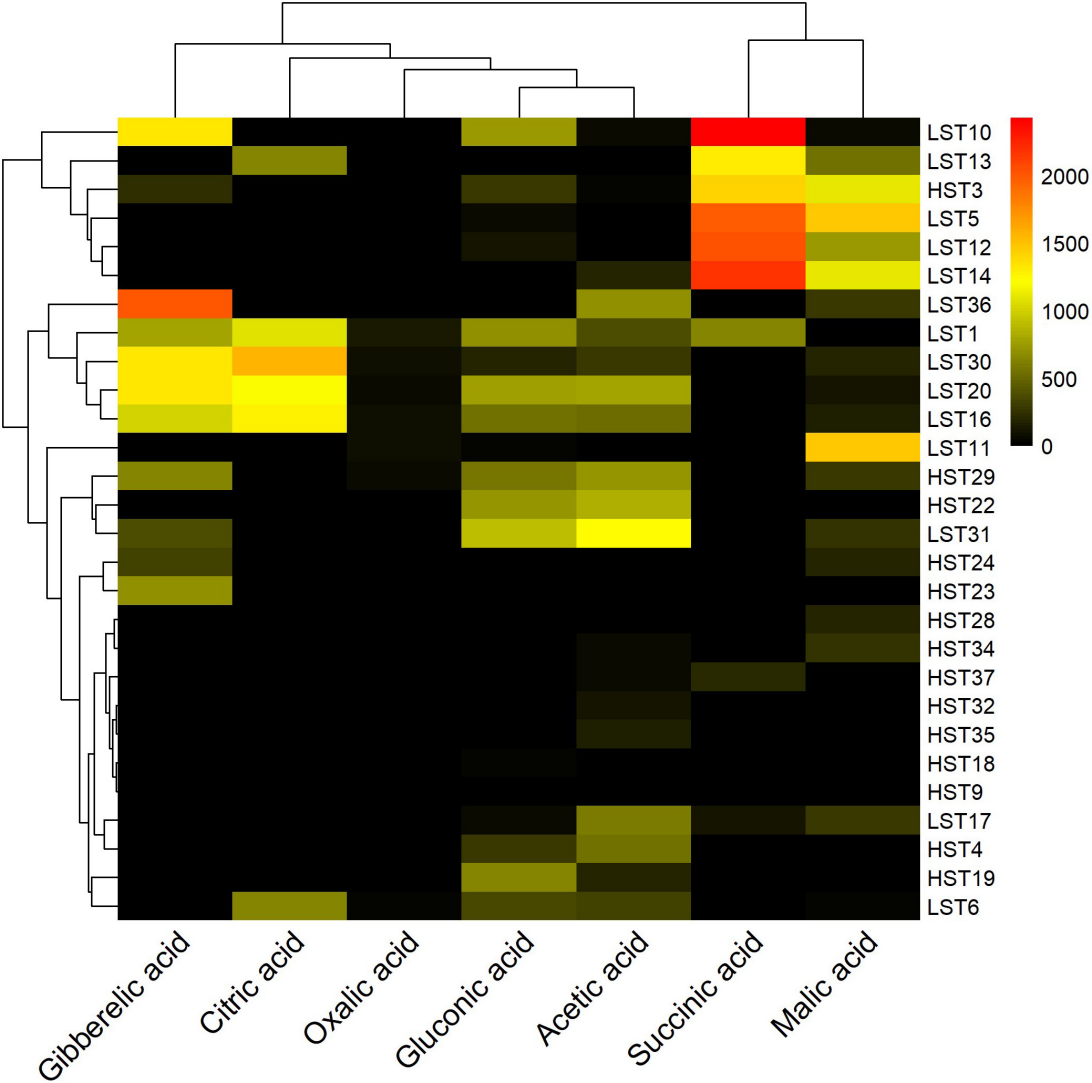
Heat map showing clusters of organic acids produced by PSB as functional indicators of bacterial phosphorus solubilization activity. Data were clustered using R package: pheatmap. Red color indicates maximum organic acid concentration, while black color indicates no organic acid production. Bacterial strains isolated from low-P soils are labeled as LST, while those isolated from high-P soils are labeled as HST.

PSB strains were grouped into distinct clusters based on their organic acid production profiles, and a heatmap was used to visualize the quantitative distribution of the various organic acids produced by the strains (Figure 5). Cluster 1, including *Enterobacter* sp. LST10, *Citrobacter* sp. LST13, *Acinetobacter* sp. HST3, *Enterobacter* sp. LST5, *Enterobacter* sp. LST12, and *Enterobacter* sp. LST14, exhibited high production of malic acid and succinic acid, ranging from 1100 to –2,000 μg/mL. Cluster 2 (*Klebsiella* sp. LST36, *Acinetobacter* sp. LST1, *Acinetobacter* sp. LST30, *Acinetobacter* sp. LST20 and *Acinetobacter* sp. LST16) showed maximum production of gibberellic acid and citric acid (1,000–2,000 μg/mL) and moderate levels of gluconic acid and acetic acid (500–1,000 μg/mL) production. Higher organic acids production by L_*PSB*_ isolates suggests organic acids secretion as a crucial P acquisition strategy of bacterial communities in P-deficient soils. Cluster 5, which comprises PSB isolated from high-P soils (*Enterobacter* sp. HST28, *Acinetobacter* sp. HST34, *Enterobacter* sp. HST37, *Franconibacter* sp. HST32, *Klebsiellla* sp. HST35, *Citrobacter* sp. HST18, and *Pantoea* sp. HST9), exhibited no or low organic acid production. This could be due to the reduced selective pressure of mineral P solubilization in high-P environments.

### 3.4 Impact of bacterial inoculation on wheat growth yield and P content

**FIGURE 6:**
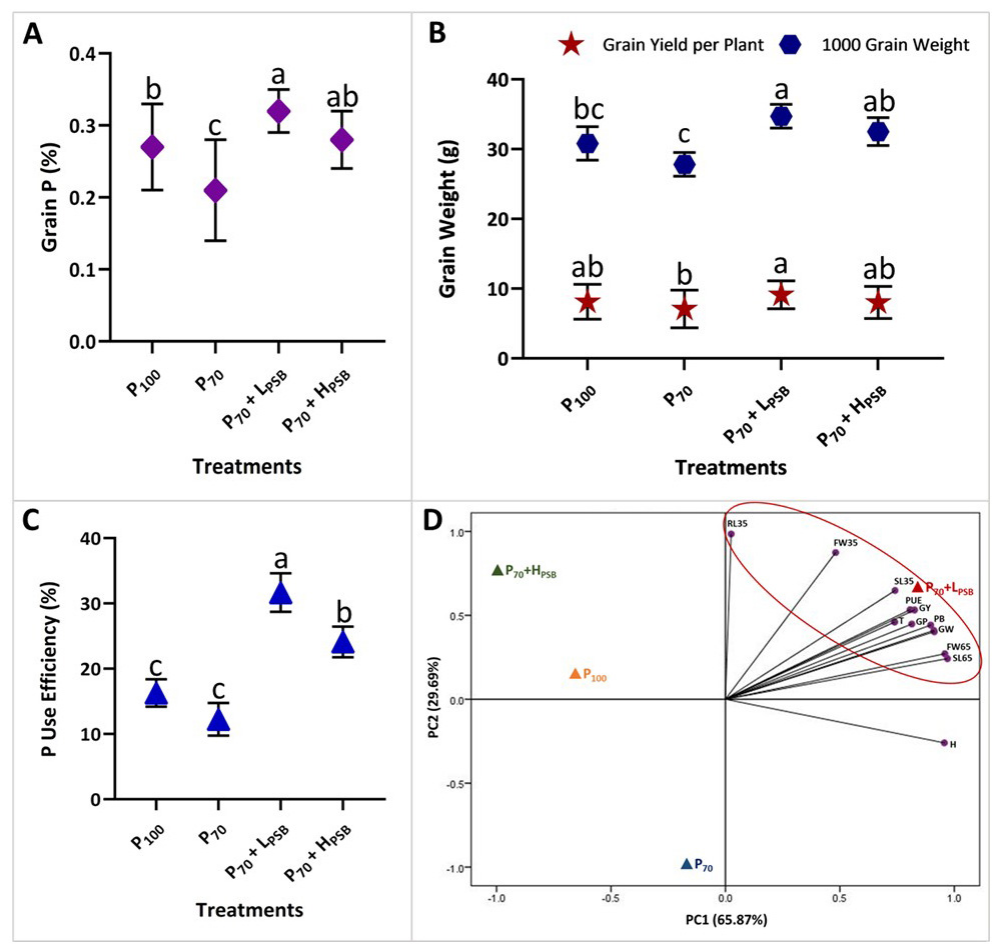
Effect of PSB inoculation on grain P content **(A)**, Grain yield per plant and 1,000 grain weight **(B)**, P use efficiency **(C)** and principal component analysis **(D)**. PCA of vegetative growth attributes, yield, and grain P in pot experiment. RL35: Root Length at35DAG, SL35: Shoot Length at 35 DAG, FW35: Fresh Weight at 35 DAG, SL65: Shoot Length at 65DAG, FW65: Fresh Weight at 65 DAG, H: Plant Height at harvest stage, PB: Plant Biomass at harvest stage, GY: Grain Yield per plant, GW: 1,000 Grain Weight, and GP: Grain P content. Values represented here are average of three biological replicates. Error bars represent standard deviation (St. Dev.). According to Least Significant Difference (LSD), means with statistically significant differences are represented by distinct letters, but statistically similar means are represented by the same letter at P = 0.05. Control-1: P_100_ (100% of recommended P dose in form of DAP), Control-2: P_70_ (70% of recommended P dose in form of DAP), P_70_ + L_PSB_ = 70% P + Consortium of PSB isolated from low-P soils, P_70_ + H_PSB_ = 70% P + Consortium of PSB isolated from high-P soils. The letter “a” denotes the highest mean value, followed by “b” and ‘c,” with combined letters (e.g., ab, bc) indicating intermediate groups that do not differ statistically at *P* = 0.05.

The bacterial consortia stimulated wheat growth (Table 3), yield and P use efficiency (Figure 6), and the Consortium-1 (L_*PSB*_) had a stronger impact than the Consortium-2. Co-application of P_70_ and Consortium-1 L_*PSB*_ significantly enhanced shoot length by 23.5 and 23.1%, root length by 34.7 and 44.3%, as well as fresh weight by 63.2 and 57.5% at 35 and 65 days after germination DAG, respectively, compared to the control P_70_. At harvest, the more pronounced response of L_*PSB*_ was 14.1 and 24.3% increases in plant height and biomass, respectively, in comparison to P_70_. Co-application of P_70_ and Consortium-2 H_*PSB*_ significantly increased root length by 16.1 and 28%, at 35 and 65 DAG, compared to the control P_70_. H_*PSB*_ also increased plant height and fresh weight by 18.4 and 43% at 65 DAG. At harvest, H_*PSB*_ increased plant height by 11.3%. Though H_*PSB*_ inoculation had a positive impact on wheat growth as compared to control P_70_ but the effect of L_*PSB*_ application was more pronounced than H_*PSB*_. The substantial increase in vegetative growth attributes at early growth stages suggests an early stimulation of plant vigor and metabolic activity, while long-term beneficial effects of PSB inoculation are further evident from the increase in biological yield at harvest stage (Elhaissoufi et al., 2023).

**TABLE 3:** Effect of PSB inoculation on wheat growth and yield at different growth stages in pot experiment.

	At 35 DAG			At 65 DAG			At Harvest stage		
Treat-ments	Shoot length (cm)	Root length (cm)	Fresh weight (g)	Shoot length (cm)	Root length (cm)	Fresh weight (g)	Plant height (cm)	No. of tillers	Plant biomass (g)
Control-1 = P_100_	29.30 ± 4.66 b	7.30 ± 1.27 bc	0.61 ± 0.05 b	68.13 ± 3.20 b	11.70 ± 0.56 b	15.27 ± 0.55 b	78.73 ± 1.63 a	6 ± 1.00 a	20.70 ± 1.65 a
Control-2 = P_70_	28.17 ± 1.33 b	6.63 ± 0.32 c	0.57 ± 0.04 b	59.87 ± 1.95 c	9.63 ± 0.61 c	11.13 ± 1.31 c	71.00 ± 1.80 b	5 ± 1.00 a	17.76 ± 0.46 b
P_70_ + L_PSB_	34.80 ± 3.72 a	8.93 ± 0.49 a	0.93 ± 0.07 a	73.73 ± 2.29 a	13.90 ± 0.67 a	17.53 ± 0.47 a	81.03 ± 1.80 a	6 ± 0.58 a	22.08 ± 1.41 a
P_70_ + H_PSB_	31.47 ± 2.70 ab	7.70 ± 0.36 b	0.59 ± 0.10 b	70.90 ± 1.01 ab	12.30 ± 0.82 b	15.93 ± 0.60 b	79.05 ± 2.94 a	5 ± 0.69 a	19.73 ± 0.64 ab

Effect of bacterial Inoculation on various plant growth parameters at different growth stages in a pot experiment. Values represented here are average of three biological replicates, ± standard deviation (St. Dev.). According to Least Significant Difference (LSD), means with statistically significant differences are represented by distinct letters, but statistically similar means are represented by the same letter at P = 0.05. Control-1: P_100_ (100% of recommended P dose in form of DAP), Control-2: P_70_ (70% of recommended P dose in form of DAP), P_70_ + L_PSB_ = 70% P + Consortium of PSB isolated from low-P soils, P_70_ + H_PSB_ = 70% P + Consortium of PSB isolated from high-P soils. The letter “a” denotes the highest statistically significant mean value, followed by “b” and “c”, respectively.

Wheat yield traits increased by both L_*PSB*_ and H_*PSB*_ inoculation, and specifically the L_*PSB*_+P_70_ increased grain P and grain weight to higher values than observed in the Control-1 (P_100)_ treatment (Figures 6A,B). An increase of 24.54 and 28.95% was observed in 1,000 grain weight and grain yield by L_*PSB*_ inoculation, compared to Control-2 (P_70_). Higher grain yield can be correlated with high phosphorus use efficiency (PUE), which was increased by L_*PSB*_ and H_*PSB*_ inoculations to higher values than either of the Control treatments. When the growth and yield parameters were subjected to principal component analysis, it confirmed the strong but differential effects of the two bacterial consortia (Figure 6D). The treatment P_70_ + L_*PSB*_ grouped with P use efficiency, grain P, 1,000 grain weight and wheat growth parameters, underlining its strong positive impact on these parameters.

## 4 Discussion

Our results showed that in particular applying PSB isolated from low-phosphorus (P) soil improves mineral solubilization and wheat growth and yield in soils with limited P availability. We also found that a bacterial inoculant from low-phosphorus (P) soil can compensate for the need for phosphate fertilization, suggesting that it can support agricultural production. As the PSB from high-P soil were less effective, our data suggests that the bacteria from low-P soil are particularly well-adapted to acquiring mineral phosphorus through the production of organic acids. The higher release of organic acids increases proton activity, which facilitates the release of phosphorus from Ca_3_(PO_4_)_2_, thereby preventing its immobilization. Enhanced P availability has been shown to improve wheat growth attributes by promoting plant P acquisition. The results showed that the application of PSB isolated from low-P soils is an effective strategy for improving P solubilization, wheat growth and yield in a soil with limited P availability, and suggests that the bacteria may even compensate for phosphate fertilization. Improved wheat growth attributes can be linked to enhanced P availability (Karimzadeh et al., 2021), as it promotes nutrient uptake from soil by improving root system development (Liu et al., 2022) and leads to better nutrient assimilation by directly involving in photosynthesis and energy metabolism (Khan et al., 2023), resulting in overall improved plant growth.

### 4.1 Soil niche properties and bacterial abundance

We found substantial variations in soil physicochemical properties (Figure 2), indicating a moderate to strong alkaline pH range in soils across Punjab and Sindh (Figure 2A), in line with previous findings of Arain and Depar (2024), Fayyaz et al. (2025), and Liu et al. (2023). High pH could be attributed to prevailing arid and semi-arid climatic regimes, native calcareous material and low organic matter content (Bolan et al., 2023; Fayyaz et al., 2025). Soil alkalinity enhanced bacterial communities’ growth rate as reflected by high colony forming units (Supplementary Figure 1), aligning with the observations reported by Malik et al. (2018). It is plausible that the relatively high soil pH created conditions that favored a shift toward copiotrophic bacterial life strategies (Rousk et al., 2009; Wang and Kuzyakov, 2024), as alkaline environments often promote the proliferation of fast-growing microbes that utilize available nutrients rapidly. In contrast to high soil pH levels, organic matter content was found to be markedly low, ranging from 0.23 to 0.86% (Figure 2C)—below the critical threshold of 1%—providing empirical evidence of a reduction in bacterial CFUs (Supplementary Figure 1), as reported by previous studies (Stockmann et al., 2015; Yao et al., 2024). An extremely low OM content reflects intensive cultivation and high decomposition rates under arid conditions. Following the availability of P, soil organic matter was found to be the strongest predictor of culturable bacterial abundance (Supplementary Figure 1), which aligns with the findings of Bucka et al. (2021) and Yao et al. (2024). Soil organic matter content directly contributes to enhanced microbial growth by providing an enriched and improved ecological niche with higher nutrient availability. This is coupled with enhanced nutrient accessibility, improved soil texture, reduced bulk density, enhanced soil porosity and water holding capacity, and high enzyme activities (Liu et al., 2021; Qaswar et al., 2020; Sheoran et al., 2019; Sihi et al., 2017). Regarding soil nutrient status, sufficient to high potassium (K) availability was observed across the study area (Figure 2E) as reported by Wakeel (2014). This is due to the development of Pakistani soils from K-rich minerals such as mica and smectite (Wakeel and Ishfaq, 2022). As an essential macronutrient, K positively influences bacterial growth, possibly by providing a nutrient-rich niche for the bacteria. We also noted that higher bacterial counts were associated with superior P availability in K-rich soils, suggesting that improved access to essential nutrients may facilitate microbial proliferation (Garrido-Sanz et al., 2023). Among edaphic factors, soil available P was found to be the strongest predictor of bacterial growth in alkaline calcareous soils (Supplementary Figure 1). This is probably due to phosphorus forming calcium-phosphate complexes more easily under such conditions, resulting in a highly P-limited environment. Consequently, even small variations in the available pool of P in the soil strongly influence bacterial proliferation and community activity. In P-scarce environments, bacterial communities are highly sensitive to soil P availability, as evidenced by our findings and those of Makhaye et al. (2021), and responsive to exogenously applied P. In P-limited ecosystems (e.g., tropical forests), exogenous P application has been shown to enhance microbial biomass (Liu et al., 2015). Dong et al. (2015) also reported that P addition affects microbial communities by altering soil organic carbon (C), total P and available P. This added P may alter carbon allocation patterns both above- and belowground by stimulating plant growth and increasing the quantity of litter and root-derived inputs entering the soil (Feng et al., 2022). In turn, these changes in plant productivity and carbon fluxes can significantly impact the composition and functioning of soil microbial communities.

Empirical links were also established between edaphic factors and bacterial population density at a P threshold of 6.3. Here, a negative correlation was observed between soil pH and bacterial CFU, with a lower bacterial count at strongly alkaline pH (>8) (Figure 3A). The CFU decrease at strongly alkaline pH can be attributed to physiological stress that disrupts enzyme activity, membrane stability, energy metabolism and reduces solubility of essential nutrients, further limiting bacterial growth and colony formation. The high bacterial count at pH = 7.5–8 is consistent with the findings of Liu et al. (2020) and Rousk et al. (2009), and the relatively low bacterial abundance in low-P (*P* < 6.3) soils at this pH range may indicate a nutrient bottleneck. Weak positive correlation between electrical conductivity (EC) and bacterial abundance (Figure 3B) is consistent with findings of Mohammad (Mohammad, 2015), who reported no significant relationships between the EC and the soil biological parameters. However, our findings are inconsistent with those of Ma et al. (2016), who found a strong association of EC with bacterial community structure, explaining 12.5% variation in community structure. Similarly, Kim also reported distinct correlations of EC with specific bacterial phyla, positive with *Bacteroidetes* and negative with *Acidobacteria* and *Betaproteobacteria* (Kim et al., 2016), suggesting that EC exhibits diverse correlation patterns with different bacterial taxa. The weak positive correlation observed in our study may be due to culturing being used instead of metabarcoding, as in Kim et al. (2016), or reflect the coexistence of bacterial groups that show either no or weak correlation with EC. In soils with *P* > 6.3, the positive relationship between bacterial CFUs and soil organic matter content and exchangeable K, in soils with *P* > 6.3, suggests a synergistic effect of organic content and nutrient availability on bacterial growth (Figures 3C,D). The observed positive relationship between bacterial CFUs and both soil organic matter and exchangeable K in soils with available *P* > 6.3 mg kg^−1^ suggests that, once minimal P constraints are alleviated, the combined availability of organic substrates and essential cations creates a more favorable nutritional environment (Dittmann et al., 2025; Li et al., 2021). This synergy likely enhances microbial energy acquisition and cellular metabolism, thereby promoting higher bacterial growth. Furthermore, these findings may also be related to diversity parameters, given that a positive impact of organic matter and potassium on bacterial community richness has been reported elsewhere (Tian et al., 2017; Xia et al., 2024). Subject to these findings, these sites could be further explored by metabarcoding and metagenomics to enable a more comprehensive characterization of their microbial diversity and functional potential.

### 4.2 The PSB and their trait spectrum

*Acinetobacter* and *Enterobacter* PSB were particularly prevalent in the selected soils with contrasting P availabilities (Table 2). This could be linked to their ability to solubilize mineral and organic phosphate complexes by releasing organic acids and phosphatases. These two genera have previously been reported for their plant growth-promoting (PGP) traits, i.e., indole acetic acid production, zinc (Zn) solubilization, production of siderophores and organic acids (Abbas et al., 2023; Kumar et al., 2017; Mowafy et al., 2021; Mujumdar et al., 2023; Wang Y. et al., 2023). So, they mayderive benefit from these instinct traits in enhanced P solubilization and improved plant growth under recalcitrant conditions. The prevalence of Proteobacteria, particularly the Gammaproteobacteria subclass, including *Acinetobacter* and *Enterobacter* in phosphorus-rich soils of Dianchi lake area was earlier reported by Yang et al. (2012). Furthermore, metagenomics investigation confirmed the abundant occurrence of proteobacteria in agricultural soils (Wu X. et al., 2022). We also found *Klebsiella* and *Pantoea* species from a few sites, which are also previously reported as plant growth-promoting rhizobacteria (PGPR) (Bhardwaj et al., 2017; Moturu et al., 2023; Suman et al., 2020; Tariq et al., 2023). Bacterium that was originally detected as a facultatively alkaliphilic bacterial strain (Gao et al., 2017), *Franconibacter daqui* sp. was also identified in this study. Regarding PGP traits, bacterial isolates from low P soils showed high P solubilization potential, while relatively low indole acetic acid production compared to those isolated from high P soils. This supports the hypothesis of microbial response adaptation to nutrient limitation and explains the mechanism by which PSB in low-P environments prioritize processes primarily involved in phosphorus availability, i.e., the secretion of organic acids, over phytohormone synthesis. Under such P-deficient conditions, bacteria typically prioritize synthesizing and secreting organic acids, which lower the soil pH and chelate cations (e.g., Ca^2+^) that bind phosphorus. This process of acidification and chelation dissolves otherwise insoluble P minerals, thereby increasing the pool of bioavailable phosphorus (Mowafy et al., 2021; Mujumdar et al., 2023). In contrast, PSB in high P soils may allocate more metabolic resources toward the production of plant growth-promoting hormones like indole-3-acetic acid (Zaidi et al., 2009a). These contrasting functional traits suggest that soil nutrient status plays a crucial role in modifying bacterial physiology and ecological strategy. We found no comparable differences in zinc solubilization potential of bacterial isolates, either isolated from low P or high P soils. This suggests that soil P availability does not significantly regulate Zn solubilization ability of bacterial strains. Contrary to IAA production, relatively high siderophore production by PSB strains isolated from low P soils highlights the importance of siderophores in enhancing P solubility by chelation with Ca^2+^ ions and releasing bound phosphorus (Cui K. et al., 2022), thus improving ecological competitiveness of indigenous bacteria.

The mineral phosphate solubilization potential of PSB from low P soils was higher than from high P soils, as evidenced by in vitro solubilization assay of tri-calcium phosphate in comparison to those of isolated from high P soils (Figure 4). This corroborates the hypothesis that bacterial communities are driven by P deficiency to extract phosphorus from recalcitrant substrates (Turner et al., 2014). In alkaline calcareous soils, the predominant mechanism for P solubilization is organic acids secretion by PSB, lowering the soil pH and chelation with Ca^2+^ ions, resulting in release of recalcitrant P (Hussain et al., 2021). The organic acid production analysis detected three patterns (Figure 5): first with high production of malic and succinic acid, second with high levels of gibberellic and citric acid and moderate levels of gluconic and acetic acid and third with low production of gluconic and acetic acid. Members of first and second cluster were predominantly PSB from low P soils, which highlights the importance of microbial mechanisms of organic acids secretion to mine recalcitrant P in P-limited environments (Wei et al., 2018). The greater production of malic acid, succinic acid, gibberellic acid and citric acid by bacterial isolates of low-P sites suggested P-deficiency provided substantial competitive advantages to these strains. These organic acids convert fixed P into soluble form by chelating with cations, such as Ca^2+^, in calcareous soils via their carboxyl and hydroxyl groups or lowering the surrounding soil pH (Zhang et al., 2020). In contrast, PSB isolated from high-P sites exhibited lower production of these acids, reflecting reduced selective pressure for P-release strategies when phosphorus is readily available. As malic acid has strong chelation and acidification ability with a lower dissociation constant (pKa = 3.40) (Vasoya, 2020) and succinic acid has moderate P solubilization potential with relatively high pKa = 4.21 (Gong et al., 2022). This suggests that PSBs with combined production are superior in terms of phosphorus (P) release from recalcitrant phosphorus minerals. Co-production of gibberellic acid (GA) and citric acid was evidenced in the second cluster, indicating a combination of P release and plant growth promotion (Li et al., 2023; Wagh et al., 2014). By contrast, the lower production of organic acids by PSB in high-P soils suggests that bacterial communities reduce their investment in P-mobilization strategies when P is plentiful. Instead, they likely redirect metabolic resources toward promoting growth and acquiring other nutrients. This shift helps to achieve a more balanced nutrient stoichiometry within the microbial community, which is consistent with the framework proposed by Yao et al. (2018). Our findings suggest that bacteria can allocate a greater proportion of their energy and resources in growth in relatively P-rich environments. Under P-limited conditions, trade-offs in bacterial physiological traits restrict the energy available for growth activities, with metabolic investment instead being channeled to P acquisition.

### 4.3 Stimulation of wheat growth and yield by the bacteria

Net house experiment demonstrated that both L_*PSB*_ and H_*PSB*_ inoculation significantly improved wheat growth and yield, with a superior influence of L_*PSB*_ as compared to H_*PSB*_ (Table 3; Figure 6). The higher potential of L_PSB_ to solubilize phosphate complexes increased plant P availability and improved plant growth performance (Garcia-Velazquez et al., 2020). The enhanced P accessibility can be linked to acidification of the soil by the secretion of various compounds like mineral and organic acids (Chen et al., 2023), phenolic compounds, and siderophores (Cheng et al., 2023), which improved the release of P from Ca_3_(PO_4_)_2_, preventing its immobilization (Adnan et al., 2019). The L_PSB_ most likely boosted P solubilization and availability to young plants, resulting in improved growth (Abbasi et al., 2015). At early growth stages, this growth enhancement can be critical for above and belowground plant growth (Vacheron et al., 2013), which later on contributes to improved nutrient availability and potentially enhanced water uptake due to the well-developed root system (Zaidi et al., 2015). We observed that L_PSB_ inoculation exhibited considerable improvements in root and shoot growth during the early development stage. These improvements can be attributed to P-solubilizing activity of L_PSB_, which probably enhanced P availability to young seedlings, supporting rapid early growth (Zaidi et al., 2009b) and IAA production stimulated root and shoot elongation in young seedlings (Glick, 2012). Furthermore, the well-developed root system and continued P availability are responsible for the better nutrient uptake and increased plant development at 65 days after germination (Zaidi et al., 2009a). These findings are consistent with Ashok and his colleagues, who reported improved wheat productivity and growth following PSB application (Kumar et al., 2021). Similar outcomes were also reported by Wahid and his group in alkaline calcareous soils for maize and wheat (Wahid et al., 2020).

The beneficial effects of applying L_PSB_ were most evident at harvest time, when there was a considerable improvement in yield. The increased plant height and number of tillers observed in the treatment (P_70_+L_PSB_) indicated improved overall plant growth and development. Enhanced tillering is particularly important for yield potential in cereal crops (Naseem and Bano, 2014). The increase in 1,000-grain weight observed following L_PSB_ application, relative to the controls, can be attributed to more efficient translocation of photosynthates to developing grains. This effect may also reflect an enhancement of phosphorus use efficiency, facilitating better nutrient allocation during grain development (Smith et al., 2021; Zaidi et al., 2015, 2009b). These findings are consistent with previous studies that have reported positive effects of PSB on crop growth and yield (Rizvi et al., 2021; Smith et al., 2021). Overall, the application of PSB, particularly those isolated from low P soils, greatly enhanced wheat growth and yield while reducing chemical fertilizer input. The superior performance of L_PSB_ compared to H_PSB_ can be explained by their greater capacity to acidify the rhizosphere by organic acid secretion, solubilize immobilized calcium phosphate complexes in calcareous soils with high pH, and increase P availability to support wheat growth. Here, we found that selective regulation of organic acid exudation by PSB may be an important adaptation strategy to manage P resource acquisition in P-deficient environments. We therefore emphasize the significance of considering the physiological characteristics of bacterial strains when designing and developing effective bioformulations for sustainable wheat production, with the aim of improving crop productivity by enhancing P availability in low P soils. However, these results must be validated through multi-site field trials across diverse agroecological zones to promote widespread application.

## 5 Conclusion

Our findings revealed that the spatial heterogeneity in nutrient levels, particularly P, organic matter and potassium, influences bacterial ecophysiological traits in alkaline calcareous soils. Bacterial isolates in low P soils, in particular, exhibit distinct adaptive strategies, such as enhanced secretion of organic acids, including malic, succinic, citric and gibberellic acid, which confer competitive advantages under nutrient-stressed conditions and promote P mobilization from recalcitrant sources. These responses suggest that, under P-limited conditions, PSB prioritize P acquisition over growth. The superior plant growth-promoting effects of PSB isolated from low P soils highlight their particular potential as effective bio-inoculants in reducing chemical fertilizer inputs and improving P use efficiency in wheat cultivation. The co-application of a reduced dose of chemical fertilizer (70% of the recommended amount) improved vegetative growth and grain yield. This indicates that the consortium can partially replace chemical fertilizers while maintaining or enhancing productivity. Future work should establish the impact of low P soil PSB in different soils and demonstrate their potential for field application. The latter objective could build on our experience of integrating environmental data into application strategies. Multi-location field trials are needed to validate their effectiveness across diverse agroecological zones.

## Data Availability

The datasets presented in this study can be found in online repositories. The names of the repository/repositories and accession number(s) can be found in this article/Supplementary material. The NCBI accession number(s) can be found in this article, Table 2.
