# Supplementary material for: Spatial heterogeneity of soil phosphorus influencing bacterial functional adaptations in alkaline calcareous soils
Source: Front Microbiol. 2026 Jan 28;16:1720323. doi: 10.3389/fmicb.2025.1720323 (PMC12891193; doi:10.3389/fmicb.2025.1720323)
Supplement: Supplementary file 1 [file Table_1.docx]

**Graphical Abstract**


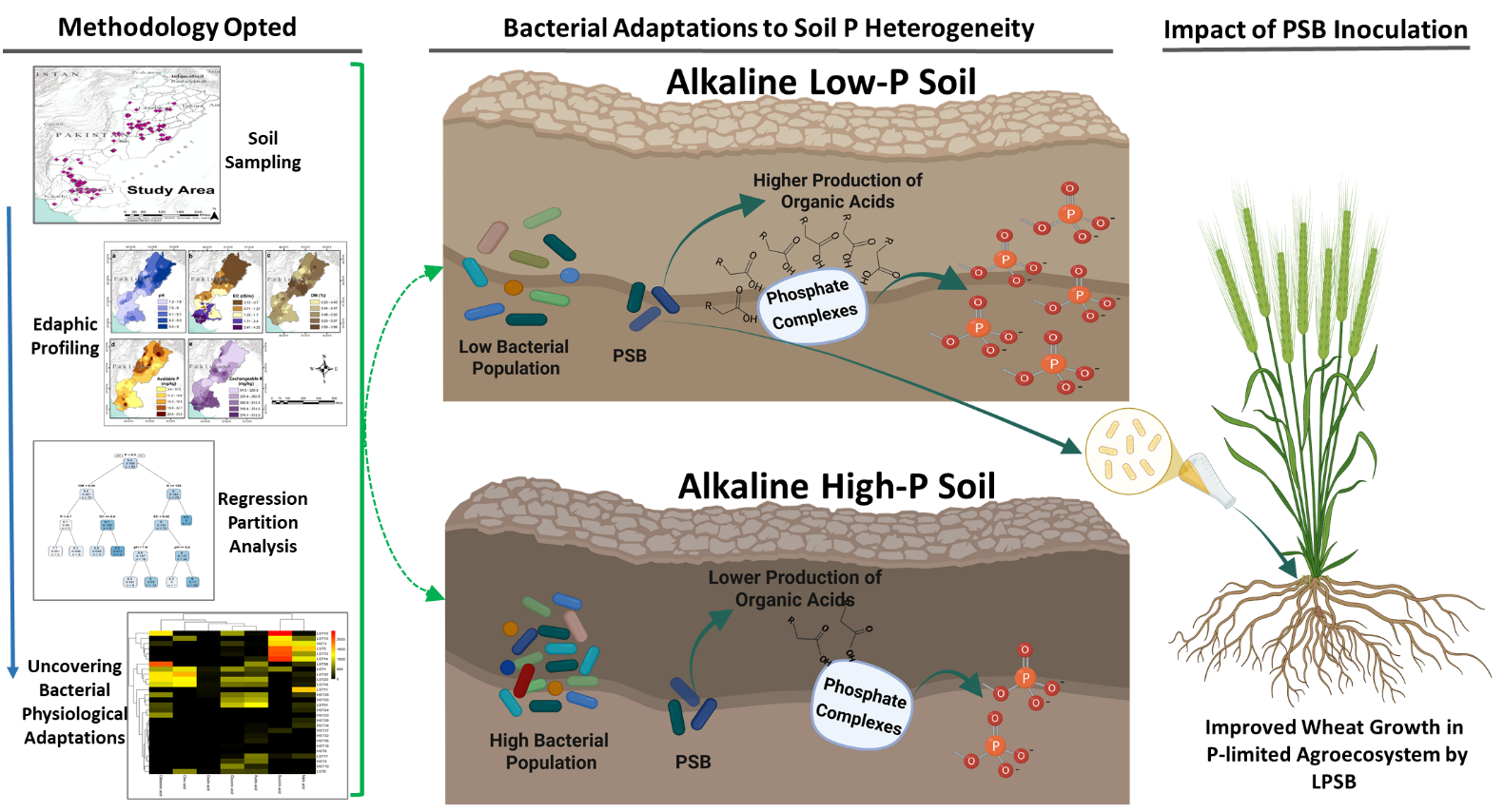


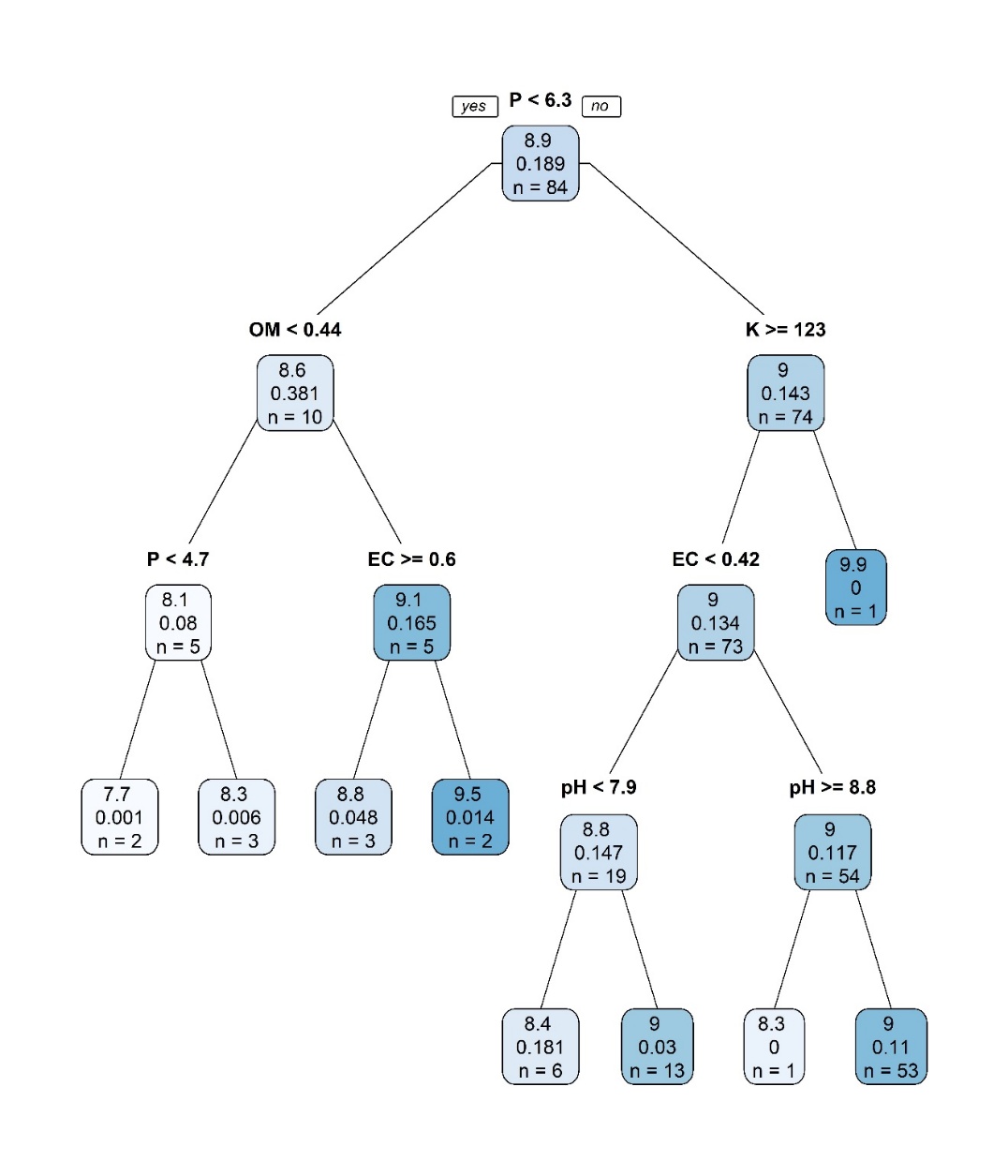


**Figure S1.** **Regression tree analysis.** This aimed to find the best partitioning parameter explaining the variance in bacterial CFU. The edaphic factors used in the analysis are following: soil pH, electrical conductivity, organic matter (OM), available phosphorus (P) and exchangeable potassium (K). Each node represents three values: 1. Bacterial log CFU, 2. Standard deviation, 3. Number of samples in this node.

**Table S1. Sampling sites and their physicochemical soil analysis used for regression partition tree analysis**

| **Serial**  **No.** | **Province** | **Longitude** | **Latitude** | **pH** | **Electrical**  **Conductivity**  **(ds/m)** | **Organic**  **Matter**  **(%)** | **Phosphorus**  **(mg/kg)** | **Potassium**  **(mg/kg)** | **Log 10**  **of CFU** |
| --- | --- | --- | --- | --- | --- | --- | --- | --- | --- |
| 1 | Punjab | 29.3208659 | 72.8737305 | 7.92 | 0.34 | 0.59 | 20.45 | 176 | 8.15 |
| 2 | Punjab | 29.3484805 | 72.9955069 | 8.06 | 0.48 | 0.42 | 4.25 | 136 | 7.69 |
| 3 | Punjab | 29.2188056 | 72.8965714 | 7.84 | 0.25 | 0.41 | 6.25 | 154 | 8.39 |
| 4 | Punjab | 29.1876804 | 72.6890285 | 8.79 | 0.79 | 0.60 | 17.55 | 308 | 8.91 |
| 5 | Punjab | 29.5255194 | 72.6768033 | 8 | 0.24 | 0.45 | 20.75 | 356 | 8.77 |
| 6 | Punjab | 29.3026418 | 71.6913798 | 7.99 | 1.49 | 0.71 | 1 | 390 | 9.00 |
| 7 | Punjab | 29.2815323 | 71.7071727 | 7.73 | 0.95 | 0.65 | 17.4 | 454 | 8.79 |
| 8 | Punjab | 29.2849799 | 71.7321035 | 8.02 | 2 | 0.58 | 9.5 | 400 | 8.61 |
| 9 | Punjab | 29.2478766 | 71.7265558 | 7.87 | 0.99 | 0.68 | 13.85 | 218 | 8.87 |
| 10 | Punjab | 30.2624521 | 71.7700630 | 7.95 | 0.3 | 0.88 | 25.4 | 212 | 8.91 |
| 11 | Punjab | 30.2363232 | 71.6882328 | 8 | 0.26 | 0.70 | 21.35 | 244 | 8.94 |
| 12 | Punjab | 29.9753492 | 71.6825251 | 7.86 | 0.75 | 0.77 | 21.15 | 234 | 8.38 |
| 13 | Punjab | 30.2464542 | 71.4804049 | 7.91 | 0.28 | 0.38 | 12.95 | 184 | 7.87 |
| 14 | Punjab | 30.1856530 | 71.5172198 | 7.83 | 0.31 | 0.15 | 5.95 | 134 | 8.22 |
| 15 | Punjab | 30.1487265 | 71.4395582 | 7.72 | 0.69 | 0.42 | 16.85 | 452 | 9.18 |
| 16 | Punjab | 28.3613257 | 70.3091972 | 7.47 | 1.24 | 0.65 | 8.95 | 192 | 9.11 |
| 17 | Punjab | 28.3819384 | 70.3353850 | 7.67 | 0.95 | 0.56 | 16.5 | 212 | 8.79 |
| 18 | Punjab | 28.3906891 | 70.3590371 | 7.72 | 2.97 | 0.59 | 6.35 | 188 | 8.94 |
| 19 | Punjab | 31.2975821 | 72.5325927 | 7.8 | 0.42 | 0.35 | 4.45 | 212 | 7.75 |
| 20 | Punjab | 31.3035798 | 72.3646283 | 7.93 | 0.23 | 0.50 | 32.1 | 180 | 8.87 |
| 21 | Punjab | 31.4279082 | 72.8844694 | 8.38 | 0.28 | 0.65 | 15.9 | 224 | 8.63 |
| 22 | Punjab | 29.9606869 | 72.0545284 | 8.24 | 0.21 | 0.35 | 6.35 | 194 | 9.00 |
| 23 | Punjab | 29.7907326 | 72.1773832 | 8.07 | 0.28 | 0.45 | 10.85 | 260 | 8.99 |
| 24 | Punjab | 29.9977058 | 72.0913699 | 8.47 | 0.32 | 0.47 | 20.25 | 338 | 8.98 |
| 25 | Punjab | 30.2624010 | 72.7387170 | 8.49 | 0.38 | 0.61 | 8.3 | 200 | 8.85 |
| 26 | Punjab | 30.1352361 | 72.6919523 | 8.8 | 1.01 | 0.58 | 13.4 | 320 | 8.35 |
| 27 | Punjab | 30.1687813 | 72.5113271 | 8.21 | 0.8 | 0.67 | 10.35 | 318 | 9.03 |
| 28 | Punjab | 29.8909511 | 72.5098139 | 8.45 | 0.21 | 0.38 | 7.7 | 122 | 9.87 |
| 29 | Punjab | 29.9330435 | 72.4971328 | 8.56 | 0.52 | 0.45 | 11.75 | 302 | 9.72 |
| 30 | Punjab | 29.9253817 | 72.4876798 | 8.41 | 0.25 | 0.60 | 4.8 | 330 | 9.63 |
| 31 | Punjab | 31.0098877 | 71.0000488 | 7.78 | 0.95 | 0.44 | 16.1 | 426 | 9.23 |
| 32 | Punjab | 30.9953908 | 71.0554630 | 7.64 | 1.33 | 0.38 | 18.2 | 280 | 9.23 |
| 33 | Punjab | 30.9952942 | 71.0547790 | 7.73 | 0.8 | 0.45 | 16.1 | 222 | 9.23 |
| 34 | Punjab | 30.0554163 | 71.1917368 | 7.77 | 0.76 | 0.67 | 21.9 | 200 | 8.84 |
| 35 | Punjab | 30.0732992 | 71.1808901 | 8.1 | 0.56 | 0.44 | 26.1 | 226 | 9.16 |
| 36 | Punjab | 30.0537850 | 71.1825300 | 7.95 | 0.34 | 0.63 | 28.1 | 144 | 9.05 |
| 37 | Punjab | 30.3162920 | 71.0668973 | 9.1 | 0.26 | 0.38 | 28.15 | 126 | 9.08 |
| 38 | Punjab | 30.5245952 | 71.5451488 | 8.47 | 0.32 | 0.44 | 5 | 178 | 8.23 |
| 39 | Punjab | 30.3373652 | 71.1961440 | 8.19 | 0.33 | 0.41 | 11.9 | 128 | 9.04 |
| 40 | Punjab | 30.3228544 | 71.8215955 | 7.78 | 0.4 | 0.44 | 32.15 | 222 | 8.71 |
| 41 | Punjab | 30.4090144 | 72.0007527 | 8.2 | 0.35 | 0.47 | 27.8 | 174 | 9.15 |
| 42 | Punjab | 29.6337024 | 70.3787238 | 8.4 | 0.57 | 0.61 | 20.05 | 378 | 9.11 |
| 43 | Punjab | 29.5439344 | 70.6132511 | 8.3 | 0.76 | 0.74 | 29.45 | 460 | 9.17 |
| 44 | Punjab | 29.1007041 | 70.3556653 | 8.31 | 0.88 | 0.55 | 9.80 | 304 | 9.22 |
| 45 | Sindh | 31.8451913 | 73.2170712 | 7.97 | 0.26 | 0.62 | 33.15 | 180 | 9.36 |
| 46 | Sindh | 29.5480970 | 71.6511847 | 7.93 | 0.3 | 0.44 | 13.85 | 184 | 7.98 |
| 47 | Sindh | 25.7943080 | 68.6752069 | 7.59 | 2.22 | 0.38 | 14.05 | 186 | 8.70 |
| 48 | Sindh | 25.7942404 | 68.6758251 | 7.8 | 1.17 | 0.50 | 6.90 | 298 | 9.36 |
| 49 | Sindh | 25.8034220 | 68.6120321 | 7.52 | 3.3 | 0.65 | 17.8 | 458 | 9.20 |
| 50 | Sindh | 25.9690718 | 68.7470201 | 7.67 | 2.84 | 0.35 | 12.30 | 550 | 9.20 |
| 51 | Sindh | 26.0596988 | 68.9777018 | 7.7 | 0.99 | 0.42 | 11.25 | 348 | 7.99 |
| 52 | Sindh | 25.8698857 | 68.6866963 | 7.81 | 0.41 | 0.59 | 15.65 | 188 | 8.90 |
| 53 | Sindh | 26.0020792 | 68.6307355 | 7.61 | 0.7 | 0.44 | 15.80 | 126 | 9.43 |
| 54 | Sindh | 26.1296303 | 68.7607120 | 7.86 | 0.96 | 0.38 | 9.55 | 316 | 8.77 |
| 55 | Sindh | 26.1705552 | 68.5163026 | 7.61 | 0.65 | 0.59 | 4.10 | 200 | 8.48 |
| 56 | Sindh | 26.5023185 | 68.2317506 | 7.59 | 1.99 | 0.44 | 20.85 | 256 | 8.15 |
| 57 | Sindh | 26.5812557 | 68.3642419 | 7.3 | 2.07 | 0.45 | 15.05 | 334 | 9.05 |
| 58 | Sindh | 26.4542685 | 68.3122274 | 7.6 | 1.55 | 0.56 | 9.20 | 186 | 9.35 |
| 59 | Sindh | 26.4538681 | 68.3125903 | 7.7 | 1.75 | 0.50 | 6.95 | 346 | 9.38 |
| 60 | Sindh | 26.4699128 | 68.3284582 | 7.89 | 3.61 | 0.44 | 8.05 | 178 | 9.25 |
| 61 | Sindh | 26.4697959 | 68.2973134 | 7.67 | 0.47 | 0.56 | 19.05 | 124 | 9.13 |
| 62 | Sindh | 26.4698411 | 68.3265539 | 7.52 | 1.2 | 0.50 | 11.85 | 286 | 8.67 |
| 63 | Sindh | 26.2319956 | 68.4062046 | 7.48 | 1.56 | 0.56 | 23.45 | 314 | 9.39 |
| 64 | Sindh | 26.7299708 | 68.1700452 | 7.51 | 2.1 | 0.55 | 10.45 | 262 | 8.82 |
| 65 | Sindh | 27.0322915 | 68.2790996 | 8.24 | 0.89 | 0.42 | 15.5 | 260 | 8.92 |
| 66 | Sindh | 26.8102369 | 67.9744136 | 8.35 | 0.88 | 0.24 | 7.85 | 238 | 8.98 |
| 67 | Sindh | 25.5467437 | 69.0916108 | 7.82 | 2.43 | 0.41 | 16.3 | 344 | 9.22 |
| 68 | Sindh | 24.8675123 | 69.3299158 | 8.2 | 1.24 | 0.44 | 4.35 | 282 | 8.85 |
| 69 | Sindh | 25.3072414 | 69.1065061 | 7.6 | 4.5 | 0.38 | 9.9 | 436 | 9.23 |
| 70 | Sindh | 25.5201739 | 69.3792972 | 7.65 | 2.99 | 0.38 | 6.9 | 426 | 9.17 |
| 71 | Sindh | 25.1698888 | 69.5683609 | 7.91 | 1 | 0.44 | 11.3 | 346 | 9.17 |
| 72 | Sindh | 25.4667154 | 68.7037200 | 7.85 | 0.61 | 0.41 | 9.65 | 282 | 9.15 |
| 73 | Sindh | 25.4539980 | 68.7293549 | 7.91 | 0.52 | 0.57 | 9.1 | 398 | 8.98 |
| 74 | Sindh | 25.3318224 | 68.7973022 | 8 | 2.73 | 0.55 | 7.3 | 280 | 8.77 |
| 75 | Sindh | 25.5305601 | 68.6832374 | 8.03 | 0.42 | 0.48 | 8.75 | 278 | 9.22 |
| 76 | Sindh | 25.3888629 | 68.4243604 | 7.9 | 4.13 | 0.47 | 12.75 | 280 | 9.23 |
| 77 | Sindh | 25.4178791 | 68.3709410 | 7.7 | 3.6 | 0.41 | 28.55 | 200 | 9.02 |
| 78 | Sindh | 25.3723365 | 68.3748766 | 7.71 | 0.61 | 0.68 | 9.3 | 320 | 9.33 |
| 79 | Sindh | 25.4342947 | 68.3295443 | 7.61 | 1.99 | 0.50 | 29.15 | 230 | 9.80 |
| 80 | Sindh | 27.7124261 | 68.6701403 | 7.93 | 0.55 | 0.43 | 16.15 | 196 | 8.41 |
| 81 | Sindh | 27.4765338 | 68.7990996 | 7.83 | 0.54 | 0.59 | 3.35 | 182 | 9.40 |
| 82 | Sindh | 27.6477993 | 68.0316014 | 7.64 | 1.57 | 0.50 | 7.45 | 278 | 9.04 |
| 83 | Sindh | 24.4389424 | 67.5440022 | 7.51 | 3.32 | 0.50 | 9.05 | 460 | 8.91 |
| 84 | Sindh | 24.7538261 | 68.5650660 | 8.01 | 4.19 | 0.38 | 28.25 | 400 | 8.86 |


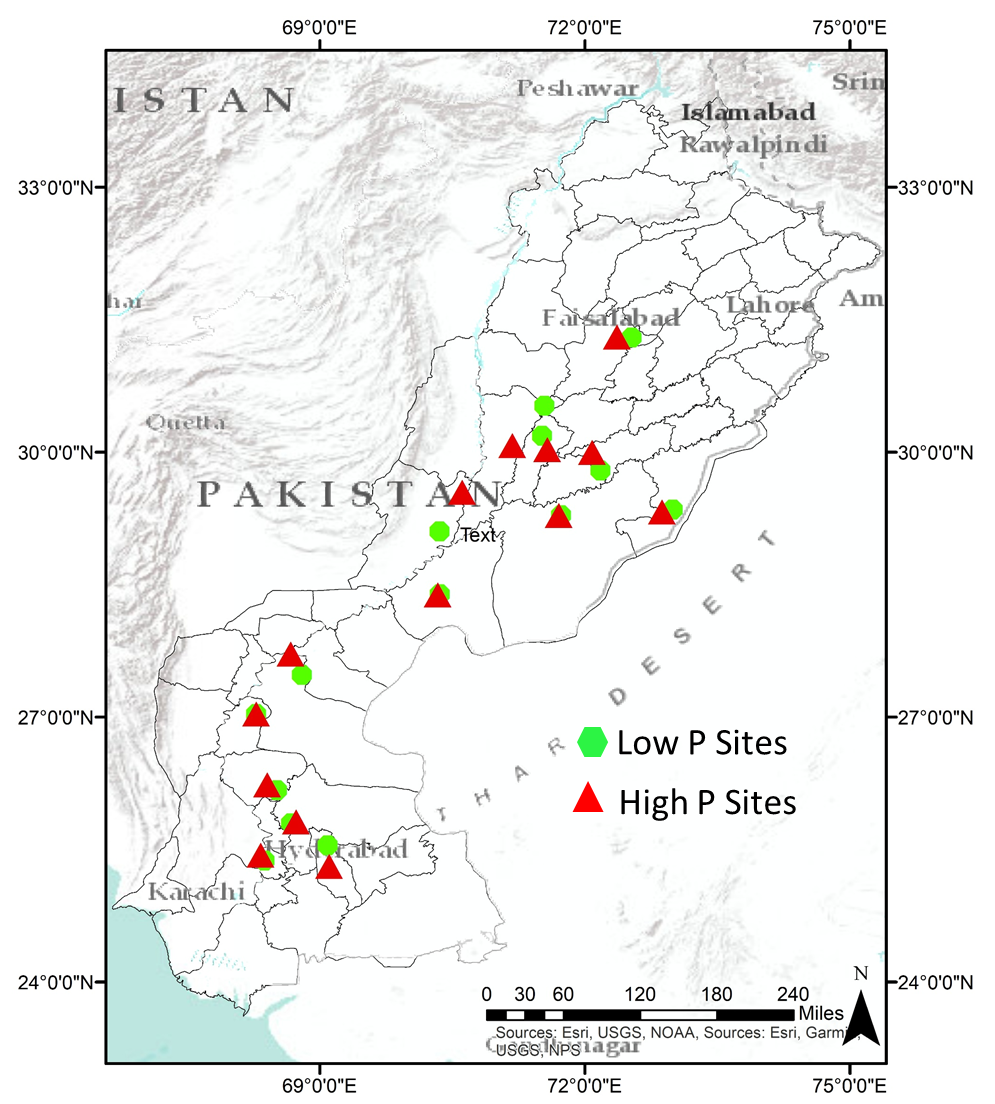


**Figure S2. Geographical distribution of selected sites with contrasting soil phosphorus availability across provinces (Punjab and Sindh) of Pakistan.**


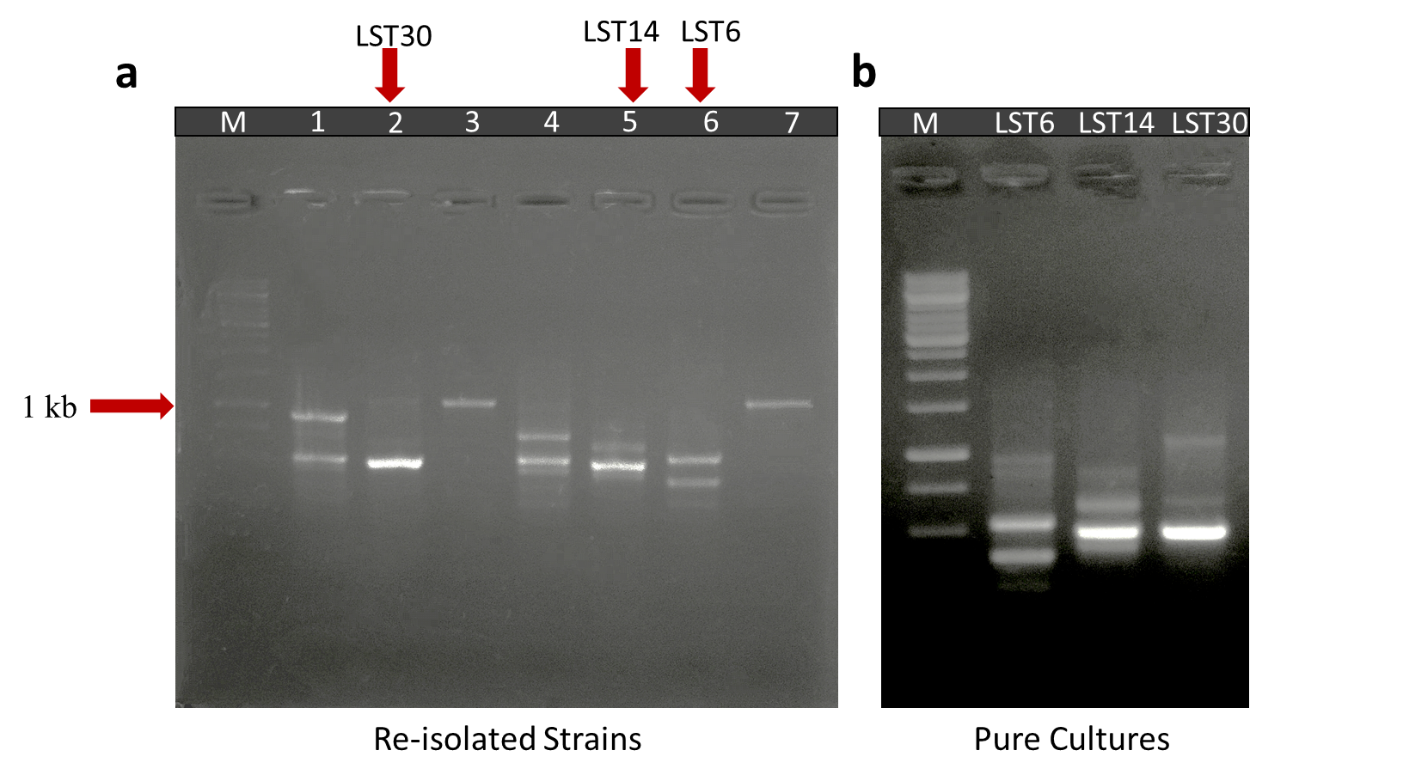


**Figure S3. BOX PCR pattern of inoculated re-isolated PSB.** Photograph of gel indicates re-isolated colonies of PSB that are morphologically similar to the inoculated consortium-1 L_PSB_, M: 1Kb DNA ladder, 1: Non-specific colony, 2: Re-isolated LST30 colony, 3,4: Non-specific colonies, 5,6: Re-isolated LST14 and LST6 colonies, 7: Non-specific colony **(a)**. Photograph of gel indicates BOX-PCR patterns of pure culture of PSB. M: 1Kb DNA ladder; LST6, LST14 andLST30 are BOX-PCR patterns of LST6, LST14 andLST30, respectively **(b)**.


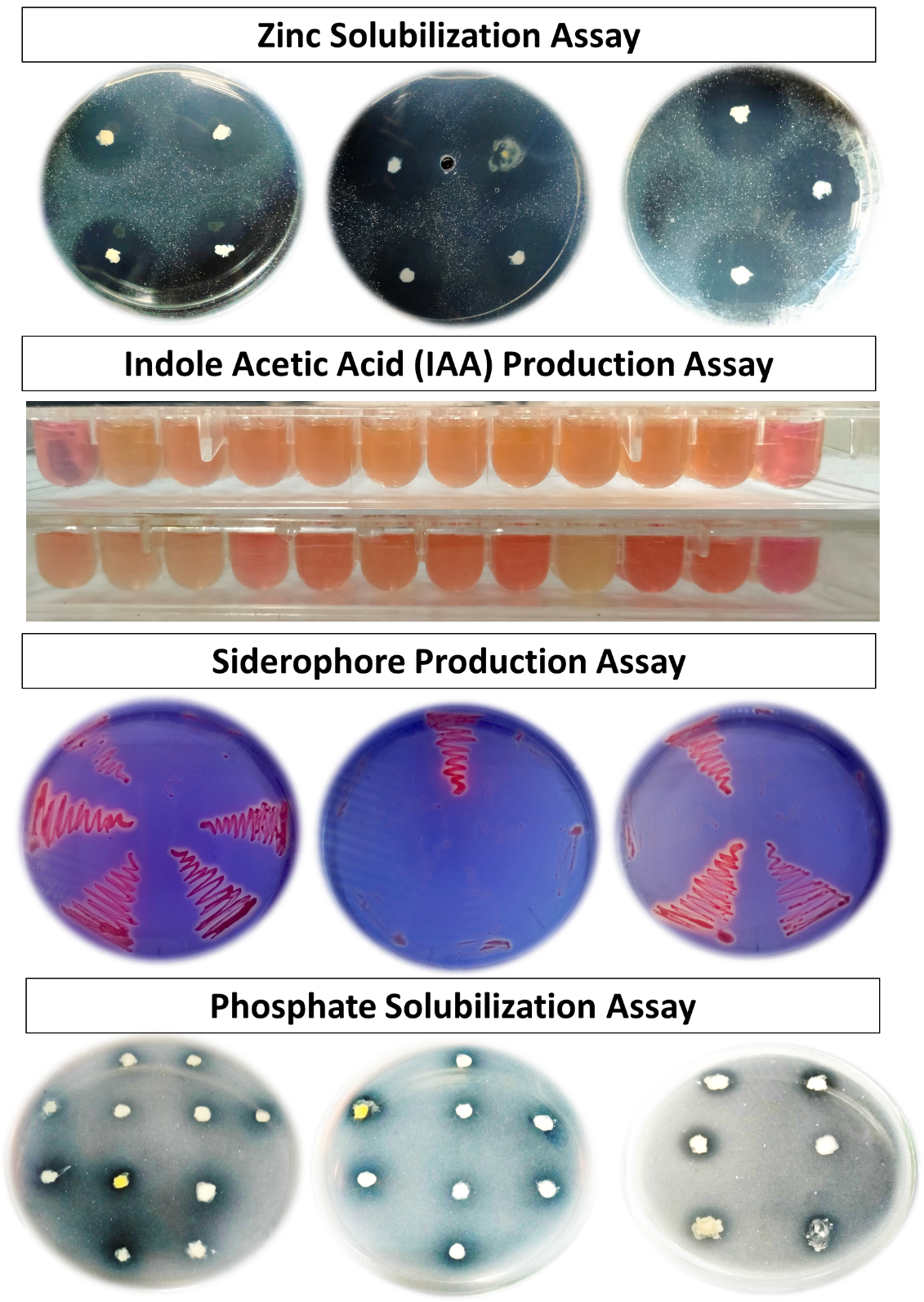


**Figure S4. Plant Growth Promoting Traits Characterization Assays of selected phosphate-solubilizing bacteria (PSB).** Zinc (Zn) solubilization was determined on Tris-minimal salts medium supplemented with zinc oxide as insoluble Zn source; the formation of clear halo zones around bacterial colonies demonstrated Zn solubilization activity. Indole-3-acetic acid production was estimated using the Salkowski method, where the pink color development indicated IAA production. Siderophore production was assessed using Chrome Azurol S (CAS) agar medium (Schwyn & Neilands, 1987), where the development of a pink halo zone around colonies indicated siderophore production activity. The phosphate (P) solubilization plate assay was performed using NBRIP agar medium having tricalcium phosphate (TCP) as an insoluble P source; the formation of clear halo zones around bacterial colonies demonstrated P solubilization activity.


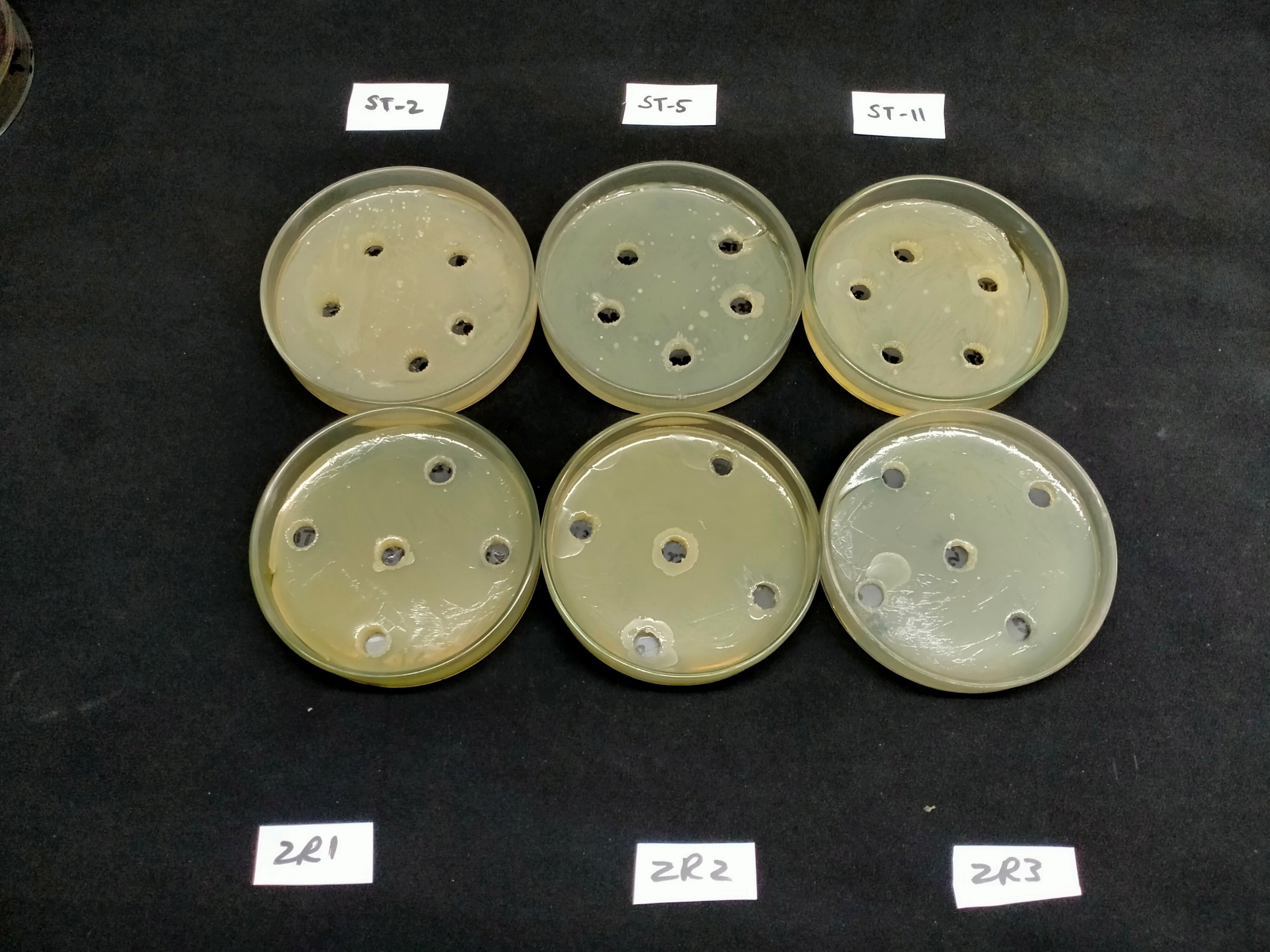


**Figure S5. Bacterial Compatibility Assay**
